# Peripheral T Cell Populations are Differentially Affected in Familial Mediterranean Fever, Chronic Granulomatous Disease, and Gout

**DOI:** 10.1007/s10875-023-01576-7

**Published:** 2023-09-16

**Authors:** Burcu Al, Mariolina Bruno, Rutger J. Röring, Simone J. C. F. M. Moorlag, Tsz Kin Suen, Viola Klück, Ruiqi Liu, Priya A. Debisarun, Orsolya Gaal, Jaydeep Bhat, Dieter Kabelitz, Frank L. van de Veerdonk, Leo A.B. Joosten, Mihai G. Netea, Katarzyna Placek

**Affiliations:** 1 Department of Immunology and Metabolism, Life and Medical Sciences Institute, University of Bonn, Bonn, Germany; 2 Department of Internal Medicine and Radboud Center for Infectious Diseases, Radboud University Medical Center, Nijmegen, Netherlands; 3 Department of Medical Genetics, Iuliu Haţieganu University of Medicine and Pharmacy, Cluj-Napoca, Romania; 4 Institute of Immunology, Christian-Albrechts-University Kiel & University Hospital Schleswig-Holstein, Campus Kiel, Kiel, Germany

**Keywords:** Familial Mediterranean fever, chronic granulomatous disease, gout, T cells, immunophenotyping, inborn errors of immunity, gamma delta T cells, flow cytometry

## Abstract

Both innate errors of immunity, such as familial Mediterranean fever (FMF) and chronic granulomatous disease (CGD), and the common inflammatory disease gout are characterized by episodes of sterile inflammatory attacks in the absence of an infection. While these disorders encompass distinct pathologies due to differentially affected metabolic pathways and inflammasome activation mechanisms, their common features are the excessive production of interleukin (IL)-1ß and innate immune cell hyperreactivity. On the other hand, the role of T cells and innate-like lymphocytes such as gamma delta (γδ) T cells in these pathologies is ill-defined. In order to widen our understanding of T cell involvement in CGD, FMF and gout pathology, we developed multicolour immunophenotyping panels for flow cytometry to characterize γδ T cells as well as CD4 and CD8 T cell populations in terms of their cytokine production, activation status, memory or naive phenotypes, exhaustion status, homing receptor expression, and cytotoxic activity. Our study is the first deep immunophenotyping analysis of T cell populations in CGD, FMF, and gout patients. We found that CGD affects the frequencies and activation status of T cells, while gout impairs the cytokine production capacity of Vδ2 T cells. FMF was characterized by decreased percentages of regulatory T cells in circulation and attenuated IFN-γ production capacity by Vδ2 T cells. Autoinflammatory syndromes and congenital defects of phagocyte differentially affect T cell compartments. Future studies are warranted to assess whether these phenotypical changes are relevant for disease pathology.

**Supplementary Information:**

The online version contains supplementary material available at 10.1007/s10875-023-01576-7.

## Introduction

Inborn errors of immunity (IEI) are caused by genetic variants which alter the function of individual genes and compromise innate and/or adaptive immunity [1]. To date, this large group of disorders encompasses 485 diseases [2]. Systemic autoinflammatory diseases (SAID), a subgroup of IEI, are described as distinct heritable disorders, mostly affecting the skin, joints, gut, and eyes, and are characterized by episodes of sterile fever and inflammation, mediated predominantly by cells and molecules of the innate immune system [3, 4]. Several SAIDs, including familial Mediterranean fever (FMF), are mediated by excessive interleukin (IL)-1β production. IL-1β maturation and secretion are under control of inflammasomes, multiprotein complexes containing the cysteine protease caspase-1 that are activated both by infections as well as endogenous stimuli (e.g., metabolic stimuli, and stress). Alterations in inflammasome activation [5] and in various metabolic pathways [6] can lead to dysregulation of IL-1β secretion and cause pathology. Gout, which is not an IEI, is another autoinflammatory disease with dysregulated IL-1β biology. Congenital defects of phagocyte are another group of IEI in which phagocytes are unable to function properly to kill the invading pathogens. CGD is one of the diseases in this subgroup with a particular defect of respiratory burst. While many of the SAIDs have been originally characterized by the absence of auto-antibodies or auto-reactive T cells, CGD-causing mutations result in the presence of lupus-like auto-antibodies and auto-reactive T cells [7–9]. Therefore, the lymphoid compartment might still be affected and contribute to the pathology of the diseases [10, 11]. Furthermore, dysregulated IL-1β production likely affects the T cell compartment in these diseases, as the effect of this cytokine on lymphocytes has been reported [12–16]. Indeed, many autoinflammatory conditions, including FMF and gout, display upregulation of T helper type 17 (Th17)-related cytokines [17–19]. Despite of this, a systematic analysis of the T cell compartment, including innate-like gamma delta (γδ) T cells, has not been performed in theis group of diseases.

Our study focuses on two diseases with known dysregulation of IL-1 cytokine production: FMF, the most prevalent monogenic autoinflammatory disease worldwide, and gout, which is the most common form of inflammatory arthritis, as well as chronic granulomatous disease (CGD) which is a rare inherited primary immunodeficiency disorder with hyperinflammatory characteristics [20–22]. FMF is recessively inherited and caused by gain-of-function mutations in the *MEFV* (Mediterranean FeVer) gene encoding the pyrin protein [23]. Pyrin is mainly expressed in innate immune cells [24] where the activated form of the protein promotes oligomerization of apoptosis-associated speck-like protein with a caspase-recruitment domain (ASC) and inflammasome formation resulting in IL-1β production [25]. Although the expression of *MEFV* gene has not been detected in T cells according to The Human Protein Atlas [26], it has been found in other source databases such as BloodSpot (https://servers.binf.ku.dk/bloodspot/). Furthermore, numerous early studies have postulated the activation of the T cell compartment during inflammatory attacks in FMF patients [10, 27]. Another inborn error of immunity, CGD, is caused by mutations in genes encoding components of the reduced nicotinamide dinucleotide phosphate (NADPH) oxidase complex: gp91^phox^, p22^phox^, p67^phox^, p40^phox^, or p47^phox^, which generates reactive oxygen species (ROS) [28, 29]. As a result, phagocytes such as neutrophils, monocytes, and macrophages cannot properly clear phagocytized microorganisms, leaving the body vulnerable to frequent infections and chronic inflammation [28]. This leads to life-threatening bacterial and fungal infections. Interestingly, ROS defects can also lead to a defective regulation of IL-1β production and granuloma formation [30]. In contrast, gout is not associated with monogenic mutations but is caused by increased concentrations of urate in the serum, which leads to the formation of monosodium urate (MSU) crystals in the joints [31]. Genetic factors, however, might play a role in the gout pathogenesis. Genome-wide association studies link high serum urate levels and gout to defective gene variants involved in the renal urate-transport system including: *ABCG2*, *SLC2A9*, *SLC17A1*, *SLC22A12*, *CGKR*, *PDZK1*, and others [32]. The pathology of gout is known to be driven mainly by innate immune cell responses, in which inflammasomes play a crucial role [33]. As such, innate immune cells produce an excess amount of IL-1β upon engulfing MSU crystals [34]. It has been shown that uric acid and MSU crystals have stimulatory effects also on T cells and can enhance T cell responses to secondary stimuli [35, 36]. Moreover infiltrated T cells were found in the tissues of gout patients [37]. The involvement of T cells in the pathogenesis of gout is however poorly known.

Therefore, the aim of our study is to assess the lymphoid compartment in patients with CGD, FMF and gout, with a focus on innate-like unconventional gammadelta (γδ) T cells as well as CD4 and CD8 conventional alphabeta (αβ) T cells.

## Methods

### Patient Recruitment

All gout, CGD, and FMF patients gave informed consent to use leftover blood for research purposes. Blood draw from healthy volunteers were approved by the Ethical Committee of the Radboud University Medical Center (no. NL32357.091.0 and no. NL42561.091.12).

### PBMCs Staining for Flow Cytometry

PBMCs were isolated by density gradient centrifugation on Pancoll (Pan Biotech). PBMCs were washed with PBS and incubated with Fc block solution (BioLegend) for 10 min. The antibody mixes (Table S1) in staining buffer (BD Bioscience) were added, and cells were incubated for 30 min at 4 °C in the dark. Samples were washed and stored at 4 °C in the dark until the reading.

### Intracellular Cytokine Staining for Flow Cytometry

PBMCs were incubated with phorbol 12-myristate 13-acetate (PMA) (50 ng/mL, Sigma-Aldrich) and ionomycin (1 μg/mL, Sigma-Aldrich) in the presence of Golgi Plug and Golgi Stop (BD Biosciences) in RPMI 1640 complete medium (10% fetal bovine serum, 1 mM sodium pyruvate (Gibco), 2 mM glutamax (Gibco), 100 U/ml Penicillin, and 100 ng/ml Streptomycin (Pan Biotech)) for 4 h at 37 °C. Then, the cells were washed with cold PBS, and Fc blocking followed by surface marker staining were performed as described above. Cells were washed with PBS and incubated in cytofix/cytoperm permeabilization solution (BD Bioscience) for 30 min at 4 °C in the dark. The cells were washed with the washing solution (BD Bioscience), and antibody mix in the washing solution was added. After 30 min of incubation at 4 °C in the dark, the cells were washed and stored at 4 °C in cell fixation solution (BD Bioscience) until analysis.

### Data Analysis

Flow cytometry data was analyzed using FlowJo (version 10.0) software. The graphs were generated using the GraphPad Prism (version 8.4.3) software. Non-parametric Mann–Whitney test was applied to calculate statistical significance. Two-tailed *p* values were considered statistically significant if below 0.05. Significant *p* values were shown with asterisks as follows: * < 0.05, ** < 0.01, *** < 0.001.

## Results

To characterize the T cell populations in patients with CGD, FMF, and gout in steady state, we applied several flow cytometry multi-color immunophenotyping panels on peripheral blood mononuclear cells (PBMCs) isolated from patients in between febrile episodes and without known ongoing infections and from healthy controls (Supplementary Table 1). We hypothesize that T cell populations can undergo phenotypical and functional changes caused by recurrent inflammation, deficiencies in metabolic pathways, or hyperuricemia in gout. T cell subpopulations were investigated in-depth for their (1) activation status and naïve/memory phenotype, (2) susceptibility to apoptosis, (3) exhaustion status, (4) cytokine production ability, (5) homing potential, (6) adhesion, and (7) cytotoxic markers.

## CGD Affects the Distribution of Peripheral Immune Cells

**Fig. 1:**
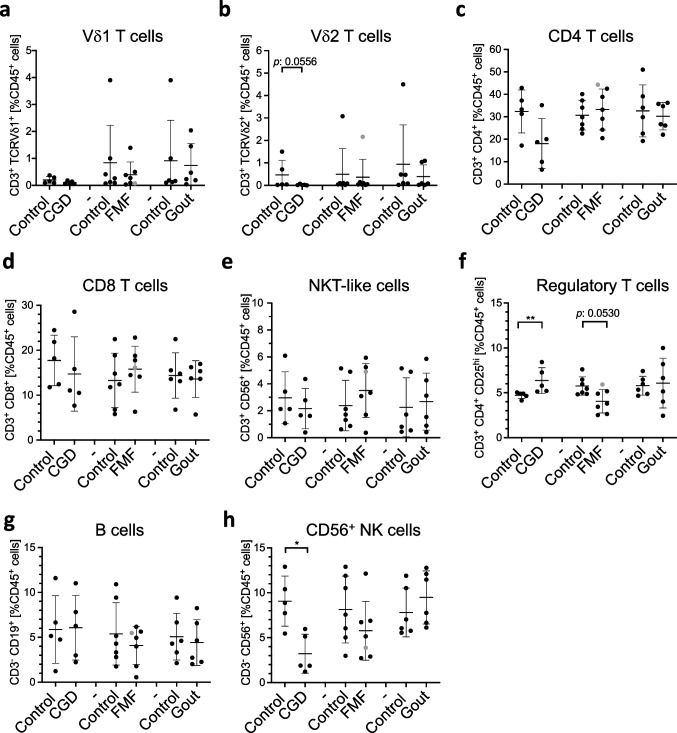
The distribution of circulating lymphocyte populations is significantly affected in CGD patients. **a**–**h** Percentages of different cell populations: CD3^+^ TCRVδ1^+^ cells (**a**), CD3^+^ TCRVδ2^+^ cells (**b**), CD3^+^ CD4^+^ cells (**c**), CD3^+^ CD8^+^ cells (**d**), CD3^+^ CD56^+^ NKT-like cells (**e**), CD3^+^ CD4^+^ CD25^hi^ CD127^−^ regulatory T cells (**f**), CD3^−^ CD19^+^ B cells (**g**), and CD3^−^ CD56^+^ NK cells (**h**) in freshly isolated PMBCs from healthy controls and patients determined by flow cytometry. CGD chronic granulomatous disease (*n* = 5), FMF familial Mediterranean fever (*n* = 7), gout (*n* = 6). Gray circle: FMF patient with Behcet disease. Non-parametric Mann–Whitney test was applied. *p ˂ 0.05, **p ˂ 0.01

First, we determined whether CGD, FMF, and gout affect immune cell distribution in peripheral blood. Our gating strategy enabled discrimination of γδ, CD4, and CD8 T cell subpopulations as well as B cells, CD56^+^ natural killer (NK) cells, regulatory T cells (Tregs), and NKT-like cells (Fig. 1 and Figure S1). Two main subsets of γδ T cells have been described in humans: Vδ1 and Vδ2, which mainly reside within epithelial tissues or are found in peripheral blood, respectively [38]. Because Vδ2 T cells are the most prevalent γδ T cell population in human peripheral blood, consisting up to 90% of the total γδ T cells [39], they were the main focus among γδ T cells in this study. We found significantly increased percentages of regulatory T cells and decreased percentages of CD56^+^ NK cells in CGD patients compared to healthy controls (Fig. 1f, h). We have also observed lower percentages of Vδ2 and CD4 T cells in CGD patients compared to healthy controls (Fig. 1b, c). However, statistical analysis did not reach significance possibly due to the small size of the cohort. The distribution of cell populations within PBMCs remained largely similar in FMF and gout patients compared to healthy controls, with the exception of almost significantly reduced percentages of regulatory T cells in FMF patients (Fig. 1f).

## Naive and Memory T Cell Compartments are Not Affected by CGD, FMF, and Gout

To further scrutinize T cell populations in CGD, FMF, and gout, we analyzed the distribution of naïve (T_Naive_), effector memory (T_EM_), central memory (T_CM_), and terminally differentiated T cells (T_EMRA_) based on CD45RA and CD27 expression (Figure S2) [40]. These distinct T cell subsets differ in their effector function such as, T_Naive_ cells (CD45RA^+^CD27^+^) do not mediate effector immune responses effectively, T_CM_ (CD45RA^−^CD27^+^) cells have a higher sensitivity to antigenic stimulation and proliferative potential, and T_EM_ (CD45RA^−^CD27^−^) cells exhibit rapid effector function and lack of proliferative capacity, while T_EMRA_ (CD45RA^+^CD27^−^) cells represent the most differentiated type of memory cells and express high levels of cytotoxic molecules [40]. We observed a large variability in distribution of different T cell subpopulations between individuals, and no significant changes could be detected between patients and healthy controls (Figure S2b, c, d). Consistently, we did not observe significant changes in the expression of other naïve and memory markers: CD127 and CD45RO, respectively (data not shown). Thus, the distribution of different effector subpopulations among T cells is not affected in gout, CGD, and FMF.

## Vδ2 and CD8 T Cells Exhibit Increased Activation Status in CGD

**Fig. 2:**
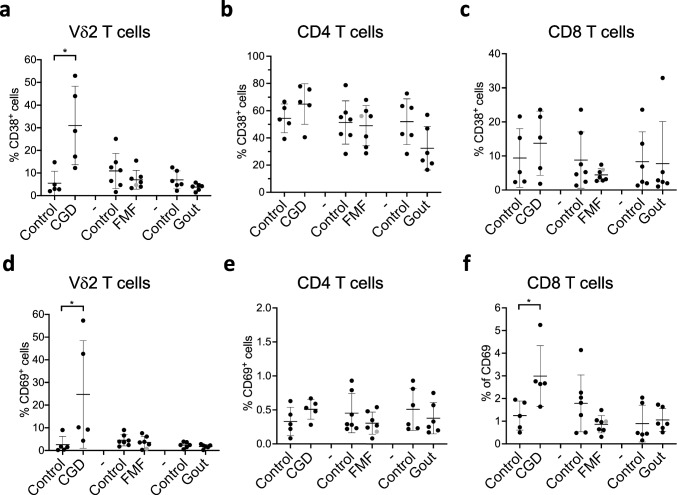
CGD results in increased expression of activation markers on Vδ2 and CD8 T cells. **a**–**f** Percentages of Vδ2 (**a**, **d**), CD4 (**b**, **e**), and CD8 T cells (**c**, **f**) expressing CD38 (**a**–**c**) and CD69 (**d**–**f**) assessed by flow cytometry on freshly isolated PMBCs from healthy controls and patients. CGD chronic granulomatous disease (*n* = 5), FMF familial Mediterranean fever (*n* = 7), gout (*n* = 6). Gray circle: FMF patient with Behcet disease. Non-parametric Mann–Whitney test was applied for statistical analysis. *p ˂ 0.05, **p ˂ 0.01

**Fig. 3:**
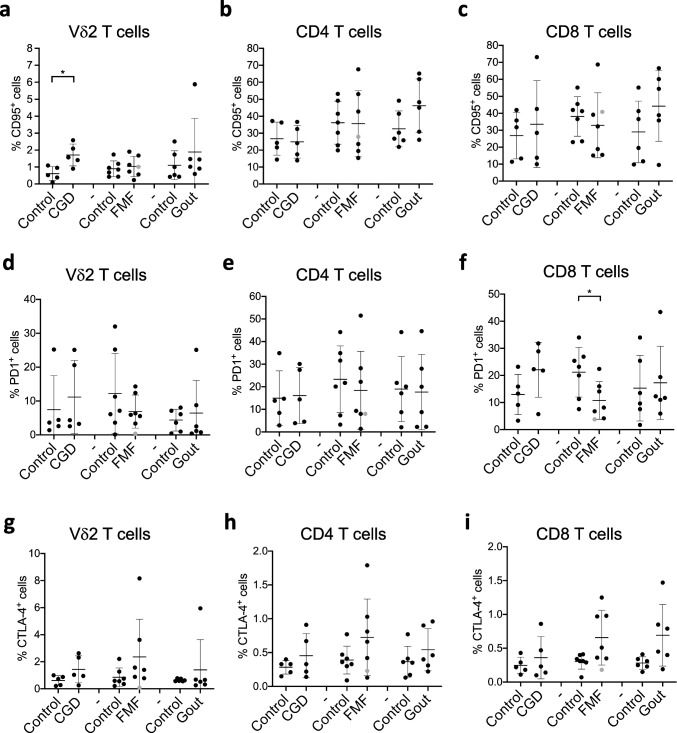
CGD and FMF affect PD-1 and CD95 expression on T cells. **a**–**i** Percentages of Vδ2 (**a**, **d**, **g**), CD4 (**b**, **e**, **h**), and CD8 T cells (**c**, **f**, **i**) expressing CD95 (**a**–**c**), PD-1 (**d**–**f**), and CTLA-4 (**g**–**i**) assessed by flow cytometry in freshly isolated PMBCs from healthy controls and patients. CGD chronic granulomatous disease (*n* = 5), FMF familial Mediterranean fever (*n* = 7), gout (*n* = 6). Gray circle: FMF patient with Behcet disease. Non-parametric Mann–Whitney test was applied for statistical analysis. *p ˂ 0.05

CD38 and CD69 are induced on T cells upon activation and are therefore commonly used as markers of activated or differentiated cells [41, 42]. We observed elevated proportions of CD38- and CD69- expressing Vδ2 T cells and CD69-expressing CD8 T cells in CGD patients (Fig. 2 and S3). Overall, this data suggests that the activation status of these T cells is increased in CGD. We further assessed the expression profile of the death receptor CD95 (Figure S4a), which is known to induce apoptosis [43]. The number of CD95-expressing Vδ2 T cells was elevated in CGD (Fig. 3a). The number of CD95-expressing CD4 and CD8 T cells was also increased in gout patients but did not reach statistical significance, possibly due to the small number of donors (Fig. 3c). Increased activation of T cells can lead to the exhaustion phenotype. Our results show that the expression of the exhaustion marker PD-1 is significantly decreased in FMF patients in comparison to healthy controls (Fig. 3f), while numbers of CTLA-4-expressing CD8 T cells have tendency to increase in FMF and gout patients (Fig. 3g–i and S3c). It is, however, important to mention that the numbers of CTLA-4- expressing cells were very low, questioning a functional relevance of this observation. This data indicates that FMF condition may affect the exhaustion status of CD8 T cells.

## Cytokine Production by Vδ2 T Cells, but Not Conventional αβ T Cells, is Impaired in Gout and FMF Patients

**Fig. 4:**
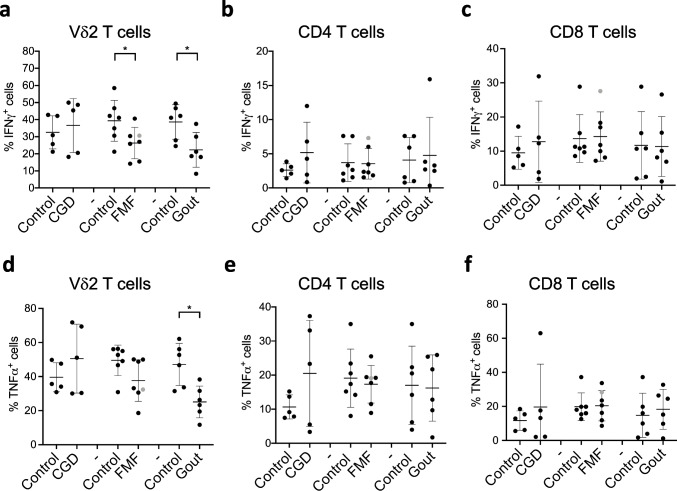
FMF and gout patients show reduced numbers of cytokine-producing Vδ2 T cells. **a**–**f** Percentages of IFN-γ- (**a**–**c**) and TNF-α- (**d**–**f**) producing Vδ2 (**a**, **d**), CD4 (**b**, **e**), and CD8 T cells (**c**, **f**) assessed by flow cytometry in freshly isolated PMBCs from healthy controls and patients. CGD cChronic granulomatous disease (*n* = 5), FMF familial Mediterranean fever (*n* = 7), gout (*n* = 6). Gray circle: FMF patient with Behcet disease. Non-parametric Mann–Whitney test was applied for statistical analysis. *p ˂ 0.05, **p ˂ 0.01

The effector function of T cells is largely driven by their cytokine production potential. Previous clinical studies demonstrated that cytokine production patterns are disrupted in patients with inborn errors of immunity, and these changes might help to distinguish different syndromes and their severity [44]. We assessed whether the capacity to produce cytokines by T cells is also affected in patients with CGD, FMF, and gout (Fig. 4). Our analysis revealed a significant reduction of IFN-γ- and TNF-α- producing Vδ2 T cells in gout (Fig. 4a, d, and S5). Furthermore, the percentages of IFN-γ-producing Vδ2 T cells were also significantly reduced in FMF patients (Fig. 4a). There were no significant changes in cytokine production among CD4 and CD8 T cells in all patients. We also analyzed the production of other cytokines, including the following: IL-4, IL-9, and IL-17α; however, we could not detect significant production of these cytokines (Figure S5c and data not shown). Thus, Vδ2 T cells are more susceptible to metabolic alterations in cytokine production than conventional T cells in examined conditions.

## CGD, FMF, and Gout Do Not Significantly Alter the Homing Receptor Expression on T Cells

**Fig. 5:**
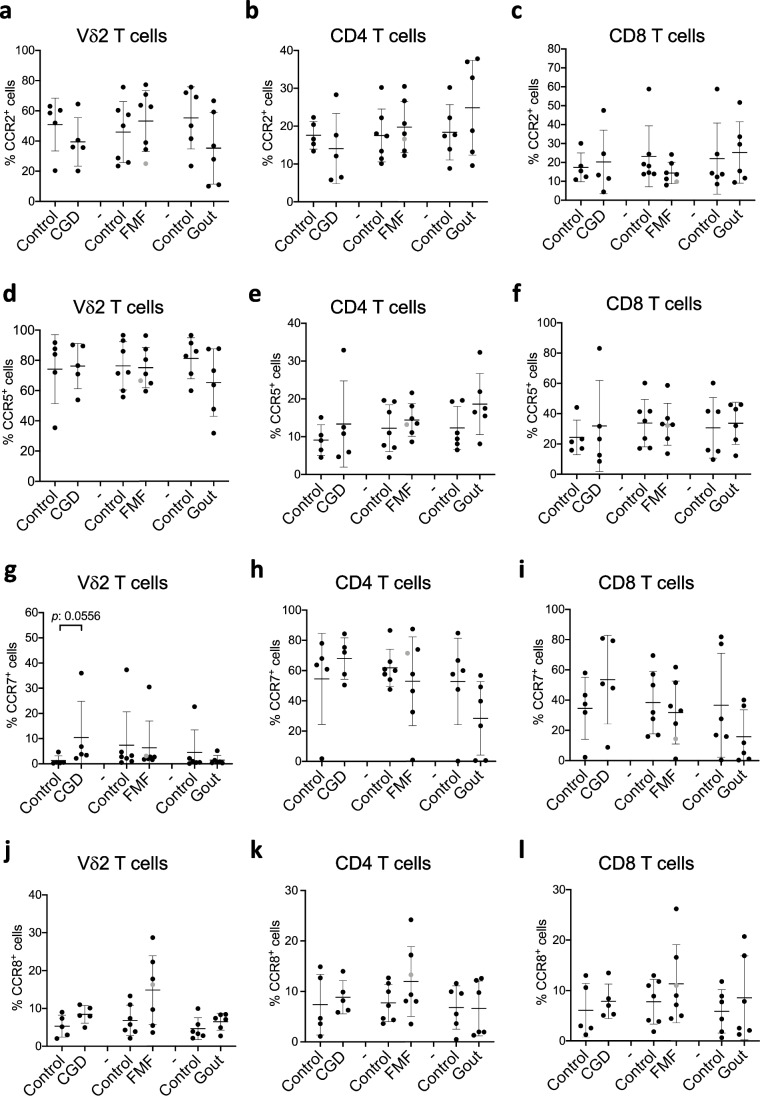
Homing receptors expression on peripheral T cells is not affected in CGD, FMF and gout. **a**–**l** Percentages of Vδ2 (**a**, **d**, **g**, **j**), CD4 (**b**, **e**, **h**, **k**), and CD8 T cells (**c**, **f**, **i**, **l**) expressing CCR2 (**a**, **b**, **c**), CCR5 (**d**, **e**, **f**), CCR7 (**g**, **h**, **i**), and CCR8 (**j**, **k**, **l**) assessed by flow cytometry in freshly isolated PMBCs from healthy controls and patients. CGD chronic granulomatous disease (*n* = 5), FMF familial Mediterranean fever (*n* = 7), gout (*n* = 6). Gray circle: FMF patient with Behcet disease. Non-parametric Mann–Whitney test was applied. *p ˂ 0.05

Immune cell migration to inflamed tissues is an important component of inflammatory processes. We therefore examined the expression profile of homing receptors to better understand the migratory status of Vδ2, CD4, and CD8 T cells in CGD, FMF, and gout (Fig. 5, S6, and S7). The expression of the following chemokine receptors was assessed by flow cytometry: CCR2 (Fig. 5a–c and S7a), which induces cell recruitment to sites of inflammation [45]; CCR5 (Fig. 5d–f and S7b), which regulates cell trafficking to the site of inflammation but also retention in tissues [46]; CCR7 (Fig. 5g–i and S7c), which mediates T cell migration from the blood to secondary lymphoid tissues [47]; CCR8 (Fig. 5j–i and S7d) and CCR4 (Figure S6a-c and S7e), which are skin homing receptors [48, 49] as well as CXCR3 (Figure S6d-f and S7f), which functions as a homing receptor to sites of infection and inflammation [50]. We observed increased numbers of CCR5-expressing CD4 T cells and reduced numbers of CCR7-expressing CD4 and CD8 T cells in gout patients (Fig. 5e, h, i). However, these numbers did not reach statistical significance, likely because of the sample size of the groups. Our results also show a trend for the increased number of CCR7^+^ Vδ2 T and CD8 T cells in CGD patients as well as the increased number of CCR8^+^ Vδ2 T, CD4 and CD8 T cells in FMF patients (Fig. 5g, i, j, k, l). Other homing receptors, CCR4 and CXCR3, did not show alterations in expression among T cell subpopulations in CGD, FMF, and gout patients compared to healthy controls (Figure S6). Altogether, no significant changes in the homing receptor expression pattern were detected in comparison to healthy controls, suggesting an unaltered migratory potential of T cells.

## The Adhesion Potential of T Cells is Not Affected by CGD, FMF, and Gout

**Fig. 6:**
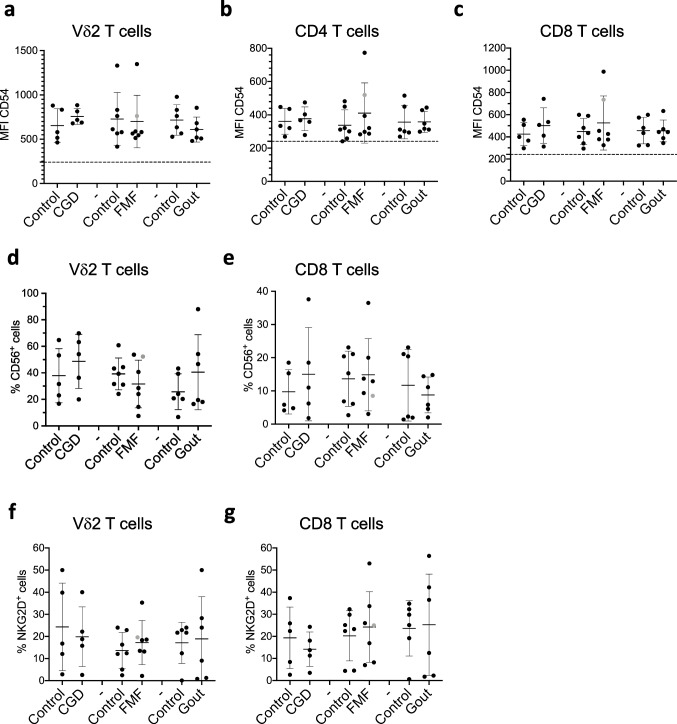
T cells exhibit no change in expression of cytotoxicity markers in FMF, CGD, and gout. **a**–**g** Expression levels of CD54 (**a**–**c**), and CD56 (**d**, **e**) and NKG2D (**f**, **g**) on peripheral Vδ2 (**a**, **d**, **f**), CD4 (**b**), and CD8 T (**c**, **e**, **g**) assessed by flow cytometry on freshly isolated PMBCs from healthy controls and patients. Dashed lines in *y*-axis show the MFI of fluorescence minus one (FMO) control. CGD chronic granulomatous disease (*n* = 5), FMF familial Mediterranean fever (*n* = 7), gout (*n* = 6). Gray circle: FMF patient with Behcet disease. Non-parametric Mann–Whitney test was applied for statistical analysis. *p ˂ 0.05, **p ˂ 0.01, ***p < 0.001

Adhesion potential of T cells is critical for cell migration, activation, and cytotoxic function. LFA-1 and CD54 interaction determines the strength and duration of cell-to-cell contact and therefore cell function [51]. This LFA-1/CD54 interaction on T cells influences their cytokine production profile, efficiency of activation, migration through tissues, and cytotoxic properties. Our assessment of LFA-1 and CD54 expression on T cells did not reveal any significant changes in all examined disorders (Fig. 6a–c, S8a-c, and S9a, d). Among T cells, CD8 and Vδ2 T cell can directly kill target cells [52, 53]. We examined whether this cytotoxic property is also affected in T cells by analyzing expression of CD56, NKG2D, and CD16 [54] (Fig. 6d–g, S8d, e, and S9b, c, e). The expression of the three molecules correlates well with each other as well as with a high content of perforin and granzyme B and cytotoxic CD8 T cell function [55, 56]. They have therefore been suggested to mark cells with cytotoxic properties. We did not observe significant differences in cell frequencies expressing the three markers between patient groups and controls (Fig. 6d–g and S8d, e).

## Discussion

In this study, we characterized three populations of blood lymphocytes: innate-like Vδ2, CD4, and CD8 T cells in CGD, FMF, and gout patients. Our comprehensive immunophenotyping revealed differential alterations in the lymphoid compartment in terms of (*i*) distribution of circulating immune cell types, and (*ii*) expression of cell surface markers and cytokines in the examined disorders.

We found that the distribution of lymphoid cells was the most affected in CGD patients, in whom CD56^+^ NK cell percentages were significantly lower and regulatory T cell percentages were significantly higher than in healthy controls (Fig. 1). Furthermore, in our study, the percentages of Vδ2 and CD4 T cell populations tend to be lower in CGD patients compared to healthy controls (Fig. 1). Previous studies reported contradictory observations, where increased, reduced, or not affected T cell numbers were shown [57–60]. The discrepancy between findings might be due to the differential treatment of patients at the time of analysis, the different distribution of patients’ age, or causative mutations in p91^phox^ or p47^phox^ coding genes within cohorts. Indeed, age-related differences in the distribution of T cells in CGD patients were reported, where individuals older than 3 years displayed reduced numbers of CD4 and CD8 T cells [57]. This is consistent with our observation that focuses on adult subjects. The cause of abrogated T cell numbers might result from the reduced proliferative capacity [59]; but whether this is intrinsic, due to deficiency of the NADPH oxidase complex, or extrinsic, as a result of disturbed immune homeostasis, remains to be determined.

T cells have been shown to express the functional phagocyte-type NADPH oxidase that is activated upon TCR stimulation [61]. Deficiency in its activity in mice results in enhanced activation of MEK-Erk pathway and augmented Th1 and Th2 responses [62] as well as reduced differentiation and suppressive activity of regulatory T cells [62, 63]. Although we did not observe significant alterations in IFN-γ production by T cells in CGD, our data revealed a trend towards increased numbers of TNF-α- and IFN-γ- producing CD4 T cells (Fig. 4), in line with mouse data [61] and with human CGD biopsies from inflamed tissues [64]. Contradictory results were found in a study of human cohort where reduced IFN-γ production by CD4 T cells, but enhanced Th17 differentiation were reported [65]. We did not find elevated numbers of IL-17α- producing T cells in CGD (Fig. 3 and data not shown). This discrepancy might originate from the different mutations causing the disease in our cohort. While Horvath et al. investigated the X-linked form of CGD, caused by p91^phox^ mutations, our cohort is mainly composed of patients with p47^phox^ mutations (Table S1). These two forms of disease might have distinct pathophysiology [66, 67]. While animal models suggest ROS-dependency of Tregs [63, 68], CGD patients with p47^phox^ mutations have been shown to have similar numbers of circulating Treg in comparison to healthy controls [68, 69]. These findings are not consistent with our observations of increased regulatory T cell numbers in CGD patients. The discrepancy in results might stem from the differences in age between the studies: our study focuses on adults (median age 36 years), while van de Geer et al. examined children (median age 13.6 years) (Table S1) [69]. A study with an increased number of inclusions will reveal if there is indeed a difference in T cell phenotype between these subgroups of patients.

Our analysis revealed an increased number of CD69-expressing CD8 and Vδ2 T cells in CGD patients (Fig. 2). CD69 is considered as an early activation and a tissue retention marker [70]. Increased expression on circulating T cells in CGD patients is less likely to be due to recent activation of these cells since the patients did not display any signs of acute attacks at the time of examination but could rather be due to the retention of these cells in the inflamed tissues. Yet, the numbers of CD69^+^ T cell are, as expected, very low within circulating lymphocytes. The significance of CD69 overexpression in CGD needs to be determined, especially at the sites of inflammatory attacks.

While most studies of CGD focus on conventional αβ T cells, this is the first report to our knowledge characterizing unconventional γδ T cell populations using human material. Apart from reduced numbers of these cells, the expression of activation markers CD38 and CD69 (Fig. 2, S3) as well as death receptor CD95 (Fig. 3a) was significantly elevated on Vδ2 T cells. This suggests an increased activation status and susceptibility to undergo apoptosis, possibly explaining the reduced numbers of Vδ2 T cells in CGD (Fig. 1b). Our analysis has also revealed a reduced number of CD56^+^ NK cells (Fig. 1h). Although early studies show a normal cytotoxic function of NK cells in CGD patients [71], a more recent cohort study revealed an association of NK cell lymphopenia with the more severe granulomas in patients with immunodeficiencies [72], indicating an involvement of these cells in granuloma formation. This suggests that development, rather than the functions of NK cells, is affected by NADPH complex deficiency. The analysis of a larger cohort is necessary to confirm these speculations.

FMF is a widely studied monogenic autoinflammatory disease, in which the T cell compartment has been best characterized so far. Early reports revealed an increased number of CD4 and CD8 T cells [10] as well as an increased number of IFN-γ-producing T cells in the asymptomatic phase and during acute inflammatory attacks [73]. The enhanced Th1 polarization in FMF patients was also suggested based on increased serum concentrations of IL-18, IL-12, and IFN-γ irrespective of the attack-free or acute inflammation phases [73–75]. However, reduced, rather than increased, IFN-γ concentrations were found in PBMC cultures from FMF patients at different stages of the disease [76]. We did not observe any significant differences in CD4 and CD8 T cell frequencies (Fig. 1) between FMF patients and healthy volunteers, but we observed lower percentages of IFN-γ-producing Vδ2 cells (Fig. 4). The discrepancy in T cell frequencies between earlier studies and our findings might be due to differences in age: while our cohort included only adults (ages 35 to 55 years old) (Table S1), earlier studies investigated pediatric patients (ages 2 to 17 years old) [10, 73]. It is well known that the T cell compartment changes during a lifespan; therefore, the differences in T cell status in children with FMF might disappear over time in adult patients due to the maturation of the T cell compartment. We observed decreased percentages of regulatory T cells in our FMF cohort compared to healthy controls, suggesting that the reduced suppressive activity of Tregs might contribute to the severity of inflammatory attacks (Fig. 1). On the other hand, a small study of 6 FMF patients [77] reported unchanged Treg frequencies in FMF patients at different stages of febrile attacks compared to the healthy control group [77] despite the increased concentration of Treg-related cytokines such as IL-10 and TGF-ß in the circulation of FMF patients [75, 78]. The discrepancy between the findings might be due to the different medication history of the patients (Table S2) or different timing of the blood sampling.

Moreover, our results suggest a reduced exhaustion status of CD8 T cells in FMF patients based on a significant decrease in the expression of PD-1 (Fig. 3). These results suggest that CD8 T cells might be more active in FMF and contribute to the inflammatory flares. Indeed, increased percentages of the activation markers CD69 and CD25 expressed on CD8 T cells were found in FMF patients during the inflammatory attack [56]. The speculation on the role of CD8 T cells in the pathology of FMF warrants further functional validation. We also found a tendency toward increased expression levels of the CCR8 homing receptor by all examined T cell populations in FMF patients (Fig. 5). CCR8 expression points to changes in the migratory patterns of T cells. Yet, the frequencies of CCR8^+^ cells in peripheral blood are very low, in accordance with other studies [79, 80] but enriched in the skin [79]. Examination of the immune cells in the skin of FMF patients will reveal whether T cells are involved in inflammatory reactions at the site of rashes, for example.

Of note, one of our FMF patients also has Behçet disease (Table S1). As this patient exhibits a huge increase in Vδ2 T cells, the observation also reported in previous studies [81] it did not cause any other outliers in our analysis. Yet, this data point needs to be interpreted with caution.

While inflammatory attacks in gout are mainly driven by cells of the innate immune system, T cells have also been found in the gouty tophi [37]. Furthermore, a recent discovery of the NLRP3 assemblage in T cells [82], which is known to be activated by MSU crystals in innate immune cells and to drive inflammatory flares in gout [33], suggests that adaptive immune cells can also be involved in gout pathology. Our analysis revealed some trends in the expression of homing receptors CCR5 and CCR7 as well as CD38 on CD4 T cells in gout patients (Figs. 5 and 2) indicating that the migratory pattern of T cells can be affected. While CCR7 regulates trafficking to lymph nodes and intestinal Peyer’s patches [83], CCR5 mediates migration and effector function of T cells to sites of inflammation [46]. Increased frequency of CCR5^+^ CD4 T cells and reduced numbers of CCR7^+^ CD4 T cells in gout (Fig. 5) suggests an enhanced recruitment of these cells to inflamed tissues. Consistently, gout patients have elevated concentrations of the CCR5 ligand: regulated upon activation normal T cell expressed, and presumably secreted (RANTES) [84–87]. CCR7 signaling has been shown to influence the Th1/Th2 balance by skewing CD4 T cell differentiation towards Th1 fate [88–91]. The Th1 cells potentiate inflammatory responses to MSU crystals [19, 92]. However, we did not find increased numbers of circulating IFN-γ^+^ T cells in gout condition (Fig. 4), consistent with recent reports [93, 94]. Similarly, we did not find significant differences in IL-17-production by T cells (data not shown), despite previous studies reporting increased levels of Th17-related cytokines in gout [18, 19]. However, our study shows significantly reduced numbers of cytokine-producing Vδ2 T cells (Fig. 4). Despite their innate-like character, γδ T cells are understudied in autoinflammatory disorders, but they are a major source of IL-17 production during the early onset of acute gout arthritis [18]. The exact role of various T helper and especially unconventional γδ T cell subsets in gout remains to be determined. Especially, further research is needed to evaluate whether the observed changes in Vδ2 T cells are due to hyperuricemia or MSU crystal deposition and how these cells act during gout flares.

As there is currently no cure for FMF, gout, and CGD current treatment strategies are aimed at reducing symptoms and preventing inflammatory attacks (in case of FMF and gout) or at preventing and managing bacterial and fungal infections and granulomas (in case of CGD). As such, the prescribed medications specifically target immunological dysfunctions, and therefore we cannot exclude the possibility that they have collateral effects on the peripheral T cell compartment. FMF and most gout patients from our cohort are on colchicine treatment which, by inhibiting the microtubule function, can affect various cell types including T cells (Table S2). Indeed, early studies reported that colchicine normalizes the CD4 to CD8 T cell ratio in FMF patients, while in healthy individuals, it reduces the total T cell numbers [95]. CGD patients, apart from receiving antibiotics (trimethoprim, sulfamethoxazole, flucloxacillin, metronidazole cream) and anti-fungals (ketoconazole, posaconazole), are also prescribed steroids (triamcinolonacetonide, prednisone, prednisolone, ciclesonide aerosole, Emovate) (Table S2). Consistent with the notion of a general immunosuppressive effect, steroids have been reported to exhibit numerous direct effects on T cells via increasing the expression of immunoregulatory proteins, inhibitory receptors, and apoptotic genes and decreasing the expression of pro-inflammatory cytokines, co-stimulatory molecules, and cell cycle mediators [96]. Other immunomodulatory medications that might influence the T cell compartment in FMF, gout, and CGD patients are IL-1β inhibitors (canakinumab or anakinra, used by two of the FMF patients) and TNF blockers (etanercept, used by one of the FMF patients), which by targeting these cytokines might modulate the polarization signals for T cells; non-steroidal anti-inflammatory drugs (NSAID) (diclofenac, ibuprofen; five FMF patients); statins (fluvastatin, simvastatin, taken by two gout patients); and allopurinol (four gout patients) (Table S2). The effect of these treatments on T cellshas either not been well investigated or has been reported contradictory. TNF blockers, for example, have been shown to either suppress cytokine production by circulating T cells in some inflammatory conditions [97–100] or to increase T cell responsiveness [101, 102]. Whereas in vitro treatment of PBMC cultures with allopurinol, a xanthine oxidase inhibitor which is used to reduce uric acid levels and treat gout, impairs cytokine production capacity by T cells [103], yet the in vivo effect is not well defined. Apart from known immunomodulators, pain medications such as opioids (oxycodone, tramadol), proton pump inhibitors reducing the amounts of stomach acid (omeprazole, pantoprazole), or even vitamins (vitamin D, folic acid) might modulate the T cell compartment in our cohort (Table S2). Yet, we cannot exclude vitamin uptake by healthy volunteers. As there is a possible impact of ongoing medical interventions on T cell populations in studied patients, it is important to report the immune aberrations in patients undergoing treatments as a potential target for further improvement of the disease burden.

Overall, our findings indicate that IEI are complex diseases of immune dysregulation in which not only myeloid but also lymphoid cell compartment is impacted. Despite the small size of our cohort, we were able to unravel significant changes in T cell populations. However, we cannot exclude the possibility that the observed alterations in the lymphoid compartment are influenced by patient treatment, genetics, clinical history, or other factors. Performing a broader examination of the T cell compartment over the course of the attack and resolution phase and at sites of inflammation, for example, synovial fluid in gout, is necessary to reveal the involvement of these cells in the pathology of the diseases. Furthermore, this is the first study to our knowledge characterizing unconventional γδ T cells in FMF and CGD patients. Our findings point to the involvement of the adaptive immune system in the pathology of certain IEI and prompt a broader assessment of T cell involvement in gout, CGD and FMF.

### Author Contribution

B.A. designed, performed, and analyzed the experiments, wrote the manuscript; M.B., R.J.R., S.J.C.F.M.M., V.K., R.L., P.A.D., O.G., L.A.B.J. and F.vdV recruited patients; T.K.S. performed and analyzed the experiments; K.P., J.B., and D.K. designed the study; L.A.B.J. and M.G.N. conceptualized the study; K.P conceptualized and supervised the study, wrote the manuscript. All authors commented on the manuscript, read and approved the final version.

### Supplementary Information

ESM 1 Supplementary Table 1 Patients and healthy controls characteristics. (a) General characteristics including sex, age, causative mutation and BMI, if applicable. (b) Group characteristics including median age, age range and female to male ratio. CGD patients 033 and 034 are twins. CGD: chronic granulomatous disease, FMF: familial Mediterranean fever, NA: not available. (PDF 88 kb)

ESM 2 Supplementary Table 2 Patients clinical data. CGD: chronic granulomatous disease, FMF: familial Mediterranean fever. NA: not available. (PDF 83 kb)

ESM 3 Supplementary Table 3 List of antibodies used for multi-color immunophenotyping flow cytometry analysis. Tagged fluorophores and corresponding vendors for each antibody are indicated. (PDF 103 kb)

ESM 4 Supplementary Figure 1 Representative core gating strategy of flow cytometry multi-color immunophenotyping panels in freshly isolated PBMCs. Duplets were excluded using FSC-H and FSC-W parameters, hematopoietic cells were gated based on CD45 expression. CD3 vs. TCR γδ gating was used to separate γδ T cells from conventional αβ T cells. TCR Vδ1 vs. TCR Vδ2 gating was applied on CD3^+^TCRγδ^+^ cells to further discriminate γδ T cell subpopulations. CD3^+^TCRγδ^-^ cells were further separated into CD4 T cells, CD8 T cells, and NKT-like cells based on CD4 vs. CD8 and CD56 expression. Regulatory T cells were identified among CD4 T cells by gating on CD25^hi^ and CD127^-^ cells. B cells and CD56^+^ NK cells were determined among CD3^-^ cells as CD19- and CD56-expressing cells, respectively. Supplementary Figure 2 Distribution of Vδ2, CD4 and CD8 T cell subsets based on naïve-effector properties assessed by flow cytometry in freshly isolated PMBCs from healthy controls and patients. (a) Representative CD45RA vs CD27 gating on Vδ2, CD4, CD8 T cells indicating distinct T cell subsets. Terminally differentiated T cells (T_EMRA_): CD45RA^+^ CD27^-^, naïve T cells: CD45RA^+^ CD27^+^, central memory T cells (T_CM_): CD45RA^-^ CD27^+^, and effector memory T cells (T_EM_): CD45RA^-^ CD27^-^. Distribution of the T cell subsets from healthy controls and patients were analyzed among Vδ2 T cells (b), CD4 T cells (c), and CD8 T cells (d) separately. CGD: chronic granulomatous disease (n=5), FMF: familial Mediterranean fever (n=7), gout (n=6) FMF patient who also has Behcet disease is indicated in lighter color. Non-parametric Mann-Whitney test was applied for statistical analysis. Supplementary Figure 3 Representative dot plots showing gating strategy for CD38 (a) and CD69 (b) on Vδ2, CD4 and CD8 T cells in freshly isolated PBMCs. Supplementary Figure 4 Representative dot plots showing gating strategy for CD95 (a) and exhaustion markers PD1 (b) and CTLA-4 (c) on Vδ2, CD4 and CD8 T cells in freshly isolated PBMCs. Supplementary Figure 5 Representative dot plots showing gating strategy for IFN-γ- (a), TNF-α- (b) and IL17-α- (c) producing Vδ2, CD4 and CD8 T cells in freshly isolated PBMCs. Supplementary Figure 6 Homing receptor expression on peripheral T cells. Percentages of CCR4- (a, b, c), and CXCR3- (d, e, f) expressing Vδ2, CD4, and CD8 T cells in freshly isolated PMBCs from healthy controls and patients. CGD: chronic granulomatous disease (n=5), FMF: familial Mediterranean fever (n=7), gout (n=6). FMF patient who also has Behcet disease is indicated in lighter color. Supplementary Figure 7 Representative dot plots showing gating strategy for CCR2 (a), CCR5 (b), CCR7 (c), CCR8 (d), CCR4 (e) and CXCR3 (f) on Vδ2, CD4 and CD8 T cells in freshly isolated PBMCs. Supplementary Figure 8 LFA-1 and CD16 expression on peripheral T cells is not affected by CGD, FMF and gout. Mean fluorescent intensity values MFI of LFA-1 (a, b, c), and cytotoxic marker CD16 (d, e) on Vδ2, CD4, and CD8 T cells assessed by flow cytometry on freshly isolated PBMCs if applicable. Dashed lines in y-axis show MFI of FMO controls. CGD: cChronic granulomatous disease (n=5), FMF: familial Mediterranean fever (n=7), gout (n=6). FMF patient who also has Behcet disease is indicated in lighter color. Non-parametric Mann-Whitney test was applied for statistical analysis. Supplementary Figure 9 Representative dot plots showing gating strategy for CD54 (a), CD56 (b) and NKG2D (c), LFA-1 (d) and CD16 (e) on Vδ2, CD4 and CD8 T cells in freshly isolated PBMC if applicable. (PDF 5221 kb)

## Data Availability

The datasets generated during and/or analyzed during the current study are available from the corresponding author on reasonable request.

## References

[1] Tangye SG, Al-Herz W, Bousfiha A, Cunningham-Rundles C, Franco JL, Holland SM (2022). Human inborn errors of immunity: 2022 update on the classification from the International Union of Immunological Societies Expert Committee. J Clin Immunol..

[2] Bousfiha A, Moundir A, Tangye SG, Picard C, Jeddane L, Al-Herz W (2022). The 2022 update of IUIS phenotypical classification for human inborn errors of immunity. J Clin Immunol..

[3] Doria A, Zen M, Bettio S, Gatto M, Bassi N, Nalotto L (2012). Autoinflammation and autoimmunity: bridging the divide. Vol. 12, Autoimmunity Reviews.

[4] Ciccarelli F, de Martinis M, Ginaldi L. An update on autoinflammatory diseases. Curr Med Chem. 2014;21(3):261–9. 10.2174/09298673113206660303. 10.2174/09298673113206660303 PMC3905709 24164192

[5] Harapas CR, Steiner A, Davidson S, Masters SL. An update on autoinflammatory diseases: inflammasomopathies. Vol. 20, Current Rheumatology Reports. Current Medicine Group LLC 1; 2018. p. 1–7. 10.1007/s11926-018-0750-4 29846819

[6] Peckham D, Scambler T, Savic S, Mc Dermott MF (2017). The burgeoning field of innate immune-mediated disease and autoinflammation. J Pathol.

[7] Olofsson P, Holmberg J, Tordsson J, Lu S, Åkerström B, Holmdahl R (2003). Positional identification of Ncf1 as a gene that regulates arthritis severity in rats. Nat Genet..

[8] Hultqvist M, Olofsson P, Holmberg J, Bäckström BT, Tordsson J, Holmdahl R (2004). Enhanced autoimmunity, arthritis, and encephalomyelitis in mice with a reduced oxidative burst due to a mutation in the Ncf1 gene. Proc Natl Acad Sci U S A..

[9] Kelkka T, Kienhöfer D, Hoffmann M, Linja M, Wing K, Sareila O (2014). Reactive oxygen species deficiency induces autoimmunity with type 1 interferon signature. Antioxid Redox Signal..

[10] Kholoussi S, Kholoussi N, Zaki ME, El-Bassyouni HT, Elnady H, Morcos B (2018). Immunological evaluation in patients with familial Mediterranean fever. Open Access Maced J Med Sci..

[11] Park H, Bourla AB, Kastner DL, Colbert RA, Siegel RM (2012). Lighting the fires within: the cell biology of autoinflammatory diseases. Nature Reviews Immunology..

[12] Koide SL, Inaba K, Steinman RM (1987). Interleukin 1 enhances T-dependent immune responses by amplifying the function of dendritic cells. J Exp Med.

[13] Van Den Eeckhout B, Tavernier J, Gerlo S. Interleukin-1 as innate mediator of T cell immunity. Vol. 11, Frontiers in immunology. NLM (Medline); 2020. p. 621931. 10.3389/fimmu.2020.621931 PMC7873566 33584721

[14] Lichtman AH, Chin J, Schmidt JA, Abbas AK (1988). Role of interleukin 1 in the activation of T lymphocytes. Proc Natl Acad Sci U S A..

[15] Ben-Sasson SZ, Hu-Li J, Quiel J, Cauchetaux S, Ratner M, Shapira I (2009). IL-1 acts directly on CD4 T cells to enhance their antigen-driven expansion and differentiation. Proc Natl Acad Sci U S A..

[16] Ben-Sasson SZ, Wang K, Cohen J, Paul WE (2013). IL-1β strikingly enhances antigen-driven CD4 and CD8 T-cell responses. Cold Spring Harb Symp Quant Biol..

[17] Galozzi P, Negm O, Bindoli S, Tighe P, Sfriso P, Punzi L (2022). A pro-inflammatory signature constitutively activated in monogenic autoinflammatory diseases. Int J Mol Sci.

[18] Liu Y, Zhao Q, Yin Y, McNutt MA, Zhang T, Cao Y (2018). Serum levels of IL-17 are elevated in patients with acute gouty arthritis. Biochem Biophys Res Commun..

[19] Yang QB, He YL, Zhang QB, Mi QS, Zhou JG (2019). Downregulation of transcription factor T-bet as a protective strategy in monosodium urate-induced gouty inflammation. Front Immunol.

[20] Xia Y, Wu Q, Wang H, Zhang S, Jiang Y, Gong T (2020). Global, regional and national burden of gout, 1990–2017: a systematic analysis of the Global Burden of Disease Study. Rheumatology.

[21] Kuo CF, Grainge MJ, Zhang W, Doherty M. Global epidemiology of gout: prevalence, incidence and risk factors. Vol. 11, Nature Reviews Rheumatology. Nature Publishing Group; 2015. p. 649–62. 10.1038/nrrheum.2015.91 26150127

[22] de Luca A, Smeekens SP, Casagrande A, Iannitti R, Conway KL, Gresnigt MS (2014). IL-1 receptor blockade restores autophagy and reduces inflammation in chronic granulomatous disease in mice and in humans. Proc Natl Acad Sci USA.

[23] Manna R, Rigante D. Familial mediterranean fever: assessing the overall clinical impact and formulating treatment plans. Vol. 11, Mediterranean Journal of Hematology and Infectious Diseases. Universita Cattolica del Sacro Cuore; 2019. 10.4084/MJHID.2019.027 PMC6548206 31205631

[24] Aksentijevich I, Centola M, Deng Z, Sood R, Balow J, Wood G (1997). Ancient missense mutations in a new member of the RoRet gene family are likely to cause familial Mediterranean fever. Cell..

[25] Heilig R, Broz P (2018). Function and mechanism of the pyrin inflammasome. Eur J Immunol [Internet].

[26] Uhlén M, Fagerberg L, Hallström BM, Lindskog C, Oksvold P, Mardinoglu A, et al. Tissue-based map of the human proteome. Science 2015;347(6220). 10.1126/science.1260419. 10.1126/science.1260419 25613900

[27] Musabak U, Sengul A, Oktenli C, Pay S, Yesilova Z, Kenar L (2004). Does immune activation continue during an attack-free period in familial Mediterranean fever?. Clin Exp Immunol.

[28] van den Berg JM, van Koppen E, Åhlin A, Belohradsky BH, Bernatowska E, Corbeel L (2009). Chronic granulomatous disease: the European experience. PLoS One..

[29] Leusen JHW, de Boer M, Bolscher BGJM, Hilarius PM, Weening RS, Ochs HD (1994). A point mutation in gp91-phox of cytochrome b558 of the human NADPH oxidase leading to defective translocation of the cytosolic proteins p47-phox and p67-phox. J Clin Investig..

[30] Holland SM. Chronic granulomatous disease. Hematology/Oncology Clinics of North America. 2013;27:89–99. 10.1016/j.hoc.2012.11.002 PMC3558921 23351990

[31] Galozzi P, Bindoli S, Doria A, Oliviero F, Sfriso P. Autoinflammatory features in gouty arthritis. Vol. 10, Journal of Clinical Medicine. MDPI; 2021. 10.3390/jcm10091880 PMC8123608 33926105

[32] Reginato AM, Mount DB, Yang I, Choi HK. The genetics of hyperuricaemia and gout. Nat Rev Rheumatol 2012;8:610–21. 10.1038/nrrheum.2012.144 PMC3645862 22945592

[33] Martinon F, Pétrilli V, Mayor A, Tardivel A, Tschopp J (2006). Gout-associated uric acid crystals activate the NALP3 inflammasome. Nature..

[34] Dalbeth N, Choi HK, Joosten LAB, Khanna PP, Matsuo H, Perez-Ruiz F (2019). Gout. Vol. 5, Nature Reviews Disease Primers.

[35] Wang B, Chen S, Qian H, Zheng Q, Chen R, Liu Y (2020). Role of T cells in the pathogenesis and treatment of gout. Vol. 88, International Immunopharmacology.

[36] Ma XJ, Tian DY, Xu D, Yang DF, Zhu HF, Liang ZH (2007). Uric acid enhances T cell immune responses to hepatitis B surface antigen-pulsed-dendritic cells in mice. World J Gastroenterol..

[37] Dalbeth N, Pool B, Gamble GD, Smith T, Callon KE, McQueen FM (2010). Cellular characterization of the gouty tophus: a quantitative analysis. Arthritis Rheum..

[38] Wu D, Wu P, Wu X, Ye J, Wang Z, Zhao S (2015). Ex vivo expanded human circulating vδ1 γδT cells exhibit favorable therapeutic potential for colon cancer. Oncoimmunology..

[39] Hoeres T, Smetak M, Pretscher D, Wilhelm M. Improving the efficiency of Vγ9Vδ2 T-cell immunotherapy in cancer. Vol. 9, Frontiers in Immunology. Frontiers Media S.A.; 2018. 10.3389/fimmu.2018.00800 PMC5916964 29725332

[40] di Mitri D, Azevedo RI, Henson SM, Libri V, Riddell NE, Macaulay R (2011). Reversible senescence in human CD4 + CD45RA + CD27 − memory T cells. J Immunol..

[41] Gorabi AM, Hajighasemi S, Kiaie N, Gheibi Hayat SM, Jamialahmadi T, Johnston TP, et al. The pivotal role of CD69 in autoimmunity. J Autoimmun. 2020;111:102453. 10.1016/j.jaut.2020.102453 32291138

[42] Piedra-Quintero ZL, Wilson Z, Nava P, Guerau-de-Arellano M. CD38: an immunomodulatory molecule in inflammation and autoimmunity. Vol. 11, Frontiers in Immunology. Frontiers Media S.A.; 2020. 10.3389/fimmu.2020.597959 PMC7734206 33329591

[43] Yamada A, Arakaki R, Saito M, Kudo Y, Ishimaru N. Dual role of Fas/FasL-mediated signal in peripheral immune tolerance. Vol. 8, Frontiers in Immunology. Frontiers Research Foundation; 2017. p. 403. 10.3389/fimmu.2017.00403 PMC5380675 28424702

[44] Galozzi P, Negm O, Greco E, Alkhattabi N, Gava A, Sfriso P (2015). Ex vivo and in vitro production of pro-inflammatory cytokines in Blau syndrome. Reumatismo.

[45] Bakos E, Thaiss CA, Kramer MP, Cohen S, Radomir L, Orr I (2017). CCR2 regulates the immune response by modulating the interconversion and function of effector and regulatory T cells. J Immunol..

[46] Oppermann M. Chemokine receptor CCR5: insights into structure, function, and regulation. Vol. 16, Cellular Signalling. Cell Signal; 2004. p. 1201–10. 10.1016/j.cellsig.2004.04.007 15337520

[47] Kobayashi D, Endo M, Ochi H, Hojo H, Miyasaka M, Hayasaka H (2017). Regulation of CCR7-dependent cell migration through CCR7 homodimer formation. Sci Rep..

[48] Yoshie O, Matsushima K (2015). CCR4 and its ligands: from bench to bedside. Int Immunol..

[49] Iellem A, Mariani M, Lang R, Recalde H, Panina-Bordignon P, Sinigaglia F (2001). Unique chemotactic response profile and specific expression of chemokine receptors CCR4 and CCR8 by CD4+CD25+ regulatory T cells. J Exp Med..

[50] Kuo PT, Zeng Z, Salim N, Mattarollo S, Wells JW, Leggatt GR (2018). The role of CXCR3 and its chemokine ligands in skin disease and cancer. Vol. 5, Frontiers in Medicine.

[51] Anderson ME, Siahaan TJ (2003). Targeting ICAM-1/LFA-1 interaction for controlling autoimmune diseases: designing peptide and small molecule inhibitors. Peptides (NY)&nbsp;.

[52] Alberts B, Johnson A, Lewis J, Raff M, Roberts K, Walter P (2002). Helper T cells and lymphocyte activation.

[53] Holtmeier W, Kabelitz D. T cells link innate and adaptive immune responses. In: Mechanisms of Epithelial Defense. Basel: KARGER; 2005. p. 151–83. 10.1159/000086659 15976493

[54] Mandelboim O, Malik P, Davis DM, Jo CH, Boyson JE, Strominger JL (1999). Human CD16 as a lysis receptor mediating direct natural killer cell cytotoxicity. Proc Natl Acad Sci USA.

[55] Pittet MJ, Speiser DE, Valmori D, Cerottini JC, Romero P (2000). Cutting edge: cytolytic effector function in human circulating CD8 + T cells closely correlates with CD56 surface expression. J Immunol..

[56] van Acker HH, Capsomidis A, Smits EL, van Tendeloo VF (2017). CD56 in the immune system: more than a marker for cytotoxicity?. Vol. 8, Frontiers in Immunology.

[57] Heltzer M, Jawad AF, Rae J, Curnutte JT, Sullivan KE (2002). Diminished T cell numbers in patients with chronic granulomatous disease. Clin Immunol..

[58] Albuquerque AS, Fernandes SM, Tendeiro R, Cheynier R, Lucas M, Silva SL (2017). Major CD4 T-cell depletion and immune senescence in a patient with chronic granulomatous disease. Front Immunol.

[59] Salmen S, Corte D, Goncalves L, Barboza L, Montes H, Al C (2007). CD40/CD40L expression in leukocytes from chronic granulomatous disease patients. APMIS [Internet].

[60] Hasui M, Hattori K, Taniuchi S, Kohdera U, Nishikawa A, Kinoshita Y (1993). Decreased CD4+CD29+ (memory T) cells in patients with chronic granulomatous disease. J Infect Dis [Internet].

[61] Jackson SH, Devadas S, Kwon J, Pinto LA, Williams MS (2004). T cells express a phagocyte-type NADPH oxidase that is activated after T cell receptor stimulation. Nat Immunol.

[62] Kwon BI, Kim TW, Shin K, Kim YH, Yuk CM, Yuk JM (2017). Enhanced Th2 cell differentiation and function in the absence of Nox2. Allergy [Internet].

[63] Efimova O, Szankasi P, Kelley TW (2011). Ncf1 (p47phox) is essential for direct regulatory T cell mediated suppression of CD4+ effector T cells. Unutmaz D, editor. PLoS One.

[64] Meda Spaccamela V, Valencia RG, Pastukhov O, Duppenthaler A, Dettmer MS, Erb J (2019). High levels of IL-18 and IFN-γ in chronically inflamed tissue in chronic granulomatous disease. Front Immunol.

[65] Horváth R, Rožková D, Lašťovička J, Poloučková A, Sedláček P, Šedivá A (2011). Expansion of T helper type 17 lymphocytes in patients with chronic granulomatous disease. Clin Exp Immunol.

[66] Magnani A, Brosselin P, Beauté J, de Vergnes N, Mouy R, Debré M (2014). Inflammatory manifestations in a single-center cohort of patients with chronic granulomatous disease. J Allergy Clin Immunol.

[67] Weening RS, Adriaansz LH, Weemaes CMR, Lutter R, Roos D (1985). Clinical differences in chronic granulomatous disease in patients with cytochrome b-negative or cytochrome b-positive neutrophils. J Pediatr..

[68] Kraaij MD, Savage NDL, Van Der Kooij SW, Koekkoek K, Wang J, Van Den Berg JM (2010). Induction of regulatory T cells by macrophages is dependent on production of reactive oxygen species. Proc Natl Acad Sci USA.

[69] van de Geer A, Cuadrado E, Slot M, van Bruggen R, Amsen D, Kuijpers T (2019). Regulatory T cell features in chronic granulomatous disease. Clin Exp Immunol.

[70] Cibrián D, Sánchez-Madrid F (2017). CD69: from activation marker to metabolic gatekeeper. Eur J Immunol.

[71] Kay HD (1983). Evidence for a nonoxidative mechanism of human natural killer (NK) cell cytotoxicity by using mononuclear effector cells from healthy donors and from patients with chronic granulomatous disease. J Immunol..

[72] Ebbo M, Gérard L, Carpentier S, Vély F, Cypowyj S, Farnarier C (2016). Low circulating natural killer cell counts are associated with severe disease in patients with common variable immunodeficiency. EBioMedicine..

[73] Aypar E, Ozen S, Okur H, Kutluk T, Besbas N, Bakkaloglu A (2003). Th1 polarization in Familial Mediterranean fever. J Rheumatol..

[74] Simsek I, Pay S, Pekel A, Dinc A, Musabak U, Erdem H (2007). Serum proinflammatory cytokines directing T helper 1 polarization in patients with familial Mediterranean fever. Rheumatol Int..

[75] Erken E, Ozer HTE, Gunesacar R (2006). Plasma interleukin-10 and interleukin-12 levels in patients with familial Mediterranean fever. Rheumatol Int..

[76] Ibrahim JN, Jounblat R, Delwail A, Abou-Ghoch J, Salem N, Chouery E (2014). Ex vivo PBMC cytokine profile in familial Mediterranean fever patients: involvement of IL-1β, IL-1α and Th17-associated cytokines and decrease of Th1 and Th2 cytokines. Cytokine..

[77] Rimar D, Rosner I, Slobodin G, Boulman N, Toubi E, Kessel A (2012). The role of regulatory T cells in familial Mediterranean fever (FMF). Clin Rheumatol..

[78] Manukyan GP, Ghazaryan KA, Ktsoyan ZA, Tatyan MV, Khachatryan ZA, Hakobyan GS (2008). Cytokine profile of Armenian patients with Familial Mediterranean fever. Clin Biochem..

[79] Schaerli P, Ebert L, Willimann K, Blaser A, Roos RS, Loetscher P (2004). A skin-selective homing mechanism for human immune surveillance T cells. J Exp Med..

[80] Ebert LM, Meuter S, Moser B (2006). Homing and function of human skin γδ T cells and NK cells: relevance for tumor surveillance. J Immunol.

[81] Hasan MS, Bergmeier LA, Petrushkin H, Fortune F (2015). Gamma delta (γδ) T cells and their involvement in Behçet’s disease.

[82] Arbore G, West EE, Spolski R, Robertson AAB, Klos A, Rheinheimer C, et al. T helper 1 immunity requires complement-driven NLRP3 inflammasome activity in CD4+ T cells. Science 2016;352(6292) 10.1126/science.aad1210. 10.1126/science.aad1210 PMC5015487 27313051

[83] Förster R, Davalos-Misslitz AC, Rot A (2008). CCR7 and its ligands: balancing immunity and tolerance. Vol. 8, Nature Reviews Immunology.

[84] Taub DD, Proost P, Murphy WJ, Anver M, Longo DL, van Damme J (1995). Monocyte chemotactic protein-1 (MCP-1), -2, and -3 are chemotactic for human T lymphocytes. J Clin Investig.

[85] Qin S, Larosa G, Campbell JJ, Smith-Heath H, Kassam N, Shi X (1996). Expression of monocyte chemoattractant protein-1 and interleukin-8 receptors on subsets of T cells: correlation with transendothelial chemotactic potential. Eur J Immunol [Internet].

[86] Diaz-Torne C, Ortiz MA, Garcia-Guillen A, Jeria-Navarro S, Sainz L, Fernandez-Sanchez S (2021). The inflammatory role of silent urate crystal deposition in intercritical gout. Rheumatology.

[87] Schall TJ, Bacon K, Toy KJ, Goeddel DV (1990). Selective attraction of monocytes and T lymphocytes of the memory phenotype by cytokine RANTES. Nature..

[88] Flanagan K, Moroziewicz D, Kwak H, Hörig H, Kaufman HL (2004). The lymphoid chemokine CCL21 costimulates naïve T cell expansion and Th1 polarization of non-regulatory CD4+ T cells. Cell Immunol..

[89] Grinnan D, Sung SS, Dougherty JA, Knowles AR, Allen MB, Rose CE (2006). Enhanced allergen-induced airway inflammation in paucity of lymph node T cell (plt) mutant mice. J Allergy Clin Immunol..

[90] Xu B, Aoyama K, Kusumoto M, Matsuzawa A, Butcher EC, Michie SA (2007). Lack of lymphoid chemokines CCL19 and CCL21 enhances allergic airway inflammation in mice. Int Immunol.

[91] Moschovakis GL, Bubke A, Dittrich-Breiholz O, Braun A, Prinz I, Kremmer E (2012). Deficient CCR7 signaling promotes T _H_ 2 polarization and B-cell activation in vivo. Eur J Immunol [Internet].

[92] Jaramillo M, Naccache PH, Olivier M (2004). Monosodium urate crystals synergize with IFN-γ to generate macrophage nitric oxide: involvement of extracellular signal-regulated kinase 1/2 and NF-κB. J Immunol.

[93] Luo G, Yi T, Zhang G, Guo X, Jiang X (2017). Increased circulating Th22 cells in patients with acute gouty arthritis. Medicine [Internet].

[94] Zhang T, Wang G, Zheng J, Li S, Xu J. Profile of serum cytokine concentrations in patients with gouty arthritis. J Int Med Res. 2021;49(11) 10.1177/03000605211055618 PMC8593300 34772308

[95] Ilfeld D, Feierman E, Kuperman O, Kivity S, Topilsky M, Netzer L (1984). Effect of colchicine on T cell subsets of healthy volunteers. Immunology.

[96] Taves MD, Ashwell JD (2021). Glucocorticoids in T cell development, differentiation and function. Vol. 21, Nature Reviews Immunology.

[97] Amital H, Barak V, Winkler RE, Rubinow A (2007). Impact of treatment with infliximab on serum cytokine profile of patients with rheumatoid and psoriatic arthritis. Annals of the New York Academy of Sciences.

[98] Pang L, Wang L, Suo T, Hao H, Fang X, Jia J (2008). Tumor necrosis factor-α blockade leads to decreased peripheral T cell reactivity and increased dendritic cell number in peripheral blood of patients with ankylosing spondylitis. J Rheumatol..

[99] Giardina AR, Accardo-Palumbo A, Ciccia F, Ferrante A, Principato A, Impastato R (2009). Blocking TNF in vitro with infliximab determines the inhibition of expansion and interferon gamma production of Vγ9/Vδ2 T lymphocytes from patients with active rheumatoid arthritis. A role in the susceptibility to tuberculosis?. Reumatismo..

[100] Popa C, Barrera P, Joosten LAB, Van Riel PLCM, Kullberg BJ, Van Der Meer JWM (2009). Cytokine production from stimulated whole blood cultures in rheumatoid arthritis patients treated with various TNF blocking agents. Eur Cytokine Netw..

[101] Zou J, Rudwaleit M, Brandt J, Thiel A, Braun J, Sieper J (2003). Up regulation of the production of tumour necrosis factor α and interferon γ by T cells in ankylosing spondylitis during treatment with etanercept. Ann Rheum Dis..

[102] Bosè F, Raeli L, Garutti C, Frigerio E, Cozzi A, Crimi M (2011). Dual role of anti-TNF therapy: enhancement of TCR-mediated T cell activation in peripheral blood and inhibition of inflammation in target tissues. Clinical Immunology..

[103] Pérez-Mazliah D, Albareda MC, Alvarez MG, Lococo B, Bertocchi GL, Petti M (2012). Allopurinol reduces antigen-specific and polyclonal activation of human T cells. Front Immunol..

