# Supplementary material for: Peripheral T Cell Populations are Differentially Affected in Familial Mediterranean Fever, Chronic Granulomatous Disease, and Gout
Source: J Clin Immunol. 2023 Sep 16;43(8):2033–48. doi: 10.1007/s10875-023-01576-7 (PMC10661758; doi:10.1007/s10875-023-01576-7)
Supplement: Supplementary file 1 — Supplementary Table 1 Patients and healthy controls characteristics. (a) General characteristics including sex, age, causative mutation and BMI, if applicable. (b) Group characteristics including median age, age range and female to male ratio. CGD patients 033 and 034 are twins. CGD: chronic granulomatous disease, FMF: familial Mediterranean fever, NA: not available. (PDF 88 kb) [file 10875_2023_1576_MOESM1_ESM.pdf]

a

	Group	Mutation	Sex	Age	BMI
**HIT_001**	control	NA	M	60	NA
**HIT_002**	control	NA	F	27	NA
**HIT_004**	Gout	NA	M	72	27,5
**HIT_006**	FMF	MEFV gene (M694I and R42W)	F	46	NA
**HIT_007**	control	NA	M	33	NA
**HIT_008**	Gout	NA	M	40	38,4
**HIT_009**	Gout	NA	M	69	35,9
**HIT_010**	CGD	p47 phox (homozygous NCF1)	M	38	21,3
**HIT_011**	CGD	p47 phox	M	36	32,2
**HIT_012**	control	NA	F	49	NA
**HIT_013**	control	NA	M	36	NA
**HIT_014**	FMF	MEFV gene (homozygous M694V)	F	45	NA
**HIT_016**	control	NA	F	54	NA
**HIT_017**	control	NA	F	31	20,5
**HIT_018**	FMF	MEFV gene (homozygous M694V)	F	35	NA
**HIT_019**	FMF	MEFV gene (M694I and R761H)	F	55	38,9
**HIT_020**	CGD	gp91 phox (splicing defect results in missing exon 7 in mRNA)	M	48	30,3
**HIT_021**	Gout	NA	M	57	28,5
**HIT_022**	Gout	NA	M	23	39,8
**HIT_024**	FMF	MEFV gene (M694V/V726A)	M	53	27,8
**HIT_025**	control	NA	F	22	NA
**HIT_026**	control	NA	M	27	NA
**HIT_027**	FMF	NA	F	46	NA
**HIT_028**	FMF	MEFV gene (M680I/E148Q)	F	43	NA
**HIT_030**	control	NA	M	26	NA
**HIT_031**	control	NA	F	30+	NA
**HIT_032**	control	NA	F	31	NA
**HIT_033**	CGD	p47 phox	M	18	21,8
**HIT_034**	CGD	p47 phox	M	18	22,8
**HIT_035**	control	NA	M	60	NA
**HIT_036**	Gout	NA	F	41	NA
**HIT_037**	control	NA	M	22	NA

b

	Total number of individuals per group	Age (median)	Age range	Female : Male ratio
**CGD Control**	5	31	22 - 49	3 : 2
**CGD**	5	36	18 - 48	0 : 5
**FMF Control**	7	47,5	31 – 60	4 : 3
**FMF**	7	46	35 - 55	6 : 1
**Gout Control**	6	31,5	26 – 60	1 :5
**Gout**	6	49	23 - 72	1 : 5

Supplementary Table 1
