# Supplementary material for: Peripheral T Cell Populations are Differentially Affected in Familial Mediterranean Fever, Chronic Granulomatous Disease, and Gout
Source: J Clin Immunol. 2023 Sep 16;43(8):2033–48. doi: 10.1007/s10875-023-01576-7 (PMC10661758; doi:10.1007/s10875-023-01576-7)
Supplement: Supplementary file 2 — Supplementary Table 2 Patients clinical data. CGD: chronic granulomatous disease, FMF: familial Mediterranean fever. NA: not available. (PDF 83 kb) [file 10875_2023_1576_MOESM2_ESM.pdf]

	Group	Comorbidity	Medication
**HIT_004**	Gout	COPID, Chronic renal failure	ETALPHA; allopurinol; ascorbic acid; chlortalidone; colecalciferole; levothyroxine; omeprazole; prednisolon; simvastatin; tiotropium; tranylcypromine; vitamin B complex
**HIT_006**	FMF	No	colchicine; macrogol; oxycodone
**HIT_008**	Gout	Eczema	allopurinol; cetomacrogol salve; ciclopirox; colchicine; ibuprofen; acetaminophen; prednisolone; quetiapine; salbutamol; spiroflor
**HIT_009**	Gout	Chronic heart failure	acenocoumarol; allopurinol; bisiprolol; colchicine; dogixine; furosemide; lisinopril; pantoprazole; acetaminophen; prednisolone; spironolactone
**HIT_010**	CGD	Ileostoma due to colitis	ketoconazole; poaconazole; triamcinolonacetonide cream; trimethoprim/sulfamethoxazole
**HIT_011**	CGD	No	alndronic acid; calcium/colecalciferol; flucloxacillin; folic acid; isotretinoin; NaCl for injection; posaconazole; prednisone; trimethiprim/sulfamethoxazole
**HIT_014**	FMF	No	Colchicine; levonorgestrel iud
**HIT_018**	FMF	No	Colchicine; colecalciferol; diclofenac; etanercept; oxycodone; pantoprazole; acetaminophen
**HIT_019**	FMF	No	canakinumab; colchicine; diclofenac; omeprazole; tramadol
**HIT_020**	CGD	Idiopatic CD4 lymphocytopenia	cetomacrogol salve; ciclesonide aerosole; lanette cream; metronidazole cream; pantoprazole; acetaminophen; posaconazole; prednisolone; salbutamol; trimethoprim/sulfamethoxazole
**HIT_021**	Gout	No	allopurinol; colchicine; prednisolone
**HIT_022**	Gout	Renal failure	alfacalcidol; benzbromaron; colchicine; colecalciferol; lisinopril; sodium hydrogencarbonate
**HIT_024**	FMF	Osteoporosis	anakinra; calcium/colecalciferol; colchicine; diclofenac-sodium; folic acid; eye salve
**HIT_027**	FMF	No	diclofenac; omeprazole
**HIT_028**	FMF	Behcet disease	colchicine; diclofenac; levothyroxine; lidocaine; pantoprazole; acetaminophen
**HIT_033**	CGD	Delayed puberty, Asthma, eczema	Emovate; folic acid; levocetirizine; posaconazole; salbutamol; triamcinolonacetonide salve; trimethoprim/sulfamethoxazole
**HIT_034**	CGD	Delayed puberty, Asthma, eczema	Emovate; folic acid; levocetirizine; posaconazole; salbutamol; triamcinolonacetonide salve; trimethoprim/sulfamethoxazole
**HIT_036**	Gout	Renal transplant	chlorhexidine cutaneous use; febuxostat; ferrofumarate; fluvastatin; labetalol; lisinopril; omeprazole; oxazepam; acetaminophen/codeine tablets; prednisolone; tacrolimus

Supplementary Table 2
