# Supplementary material for: Peripheral T Cell Populations are Differentially Affected in Familial Mediterranean Fever, Chronic Granulomatous Disease, and Gout
Source: J Clin Immunol. 2023 Sep 16;43(8):2033–48. doi: 10.1007/s10875-023-01576-7 (PMC10661758; doi:10.1007/s10875-023-01576-7)
Supplement: Supplementary file 3 — Supplementary Table 3 List of antibodies used for multi-color immunophenotyping flow cytometry analysis. Tagged fluorophores and corresponding vendors for each antibody are indicated. (PDF 103 kb) [file 10875_2023_1576_MOESM3_ESM.pdf]

Antibody	Fluorophore Name	Clone	Vendor	Location of staining
CD45	AlexaFlour 700	HI30	BioLegend	Surface
CD3	BUV395	UCHT1	BD Biosciences	Surface
CD4	PerCP-Cy5.5 or BV570	OKT4	BioLegend	Surface
CD8	BV786	SK1	BioLegend	Surface
TCRγδ	PE/Cy7	11F2	BD Biosciences	Surface
TCR Vδ2	APC or FITC	REA771	Miltenyi Biotech	Surface
TCR Vδ1	BV421		Miltenyi Biotech	Surface
TCR Vγ9	FITC	B3	BioLegend	Surface
CCR2	PE/Dazzle594	KO36C2	BioLegend	Surface
CCR4	BV510	L291H4	BioLegend	Surface
CCR5	BV421	J418F1	BioLegend	Surface
CCR7	BV605	G043H7	BioLegend	Surface
CCR8	PE	L263G8	BioLegend	Surface
CD14	BV711	M5E2	BioLegend	Surface
CD16	PerCP-Cy5.5	5.1H11	BioLegend	Surface
CD19	BV605	HIB19	BioLegend	Surface
CD25	PE/Dazzle594	M-A251	BioLegend	Surface
CD27	BV650	O323	BioLegend	Surface
CD38	BV421	HIT2	BioLegend	Surface
CD45RA	FITC	HI100	BioLegend	Surface
CD45RO	BV570	UCHL1	BioLegend	Surface
CD54	PE/Dazzle594	HA58	BioLegend	Surface
CD56	PE/Dazzle594	5.1H11	BioLegend	Surface
CD69	BV605	FN50	BioLegend	Surface
CD95	APC/Cy7	DX2	BioLegend	Surface
CD127	BV711	AO19D5	BioLegend	Surface
CTLA-4	APC/Fire750	L3D10	BioLegend	Surface
CXCR3	APC/Fire750	G043H7	BioLegend	Surface
LFA-1	BV421	m24	BioLegend	Surface
NKG2D	PE	1D11	BioLegend	Surface
PD-1	PE	EH12.2H7	BioLegend	Surface
IFN-γ	BV711	4S.B3	BioLegend	Intracellular
TNF-α	AlexaFlour 700	Mab11	BioLegend	Intracellular
IL-17α	BV421	BL168	BioLegend	Intracellular

Supplementary Table 3
