# Supplementary material for: Peripheral T Cell Populations are Differentially Affected in Familial Mediterranean Fever, Chronic Granulomatous Disease, and Gout
Source: J Clin Immunol. 2023 Sep 16;43(8):2033–48. doi: 10.1007/s10875-023-01576-7 (PMC10661758; doi:10.1007/s10875-023-01576-7)
Supplement: Supplementary file 4 — Supplementary Figure 1 Representative core gating strategy of flow cytometry multi-color immunophenotyping panels in freshly isolated PBMCs. Duplets were excluded using FSC-H and FSC-W parameters, hematopoietic cells were gated based on CD45 expression. CD3 vs. TCR γδ gating was used to separate γδ T cells from conventional αβ T cells. TCR Vδ1 vs. TCR Vδ2 gating was applied on CD3+TCRγδ+ cells to further discriminate γδ T cell subpopulations. CD3+TCRγδ- cells were further separated into CD4 T cells, CD8 T cells, and NKT-like cells based on CD4 vs. CD8 and CD56 expression. Regulatory T cells were identified among CD4 T cells by gating on CD25hi and CD127- cells. B cells and CD56+ NK cells were determined among CD3- cells as CD19- and CD56-expressing cells, respectively. Supplementary Figure 2 Distribution of Vδ2, CD4 and CD8 T cell subsets based on naïve-effector properties assessed by flow cytometry in freshly isolated PMBCs from healthy controls and patients. (a) Representative CD45RA vs CD27 gating on Vδ2, CD4, CD8 T cells indicating distinct T cell subsets. Terminally differentiated T cells (TEMRA): CD45RA+ CD27-, naïve T cells: CD45RA+ CD27+, central memory T cells (TCM): CD45RA- CD27+, and effector memory T cells (TEM): CD45RA- CD27-. Distribution of the T cell subsets from healthy controls and patients were analyzed among Vδ2 T cells (b), CD4 T cells (c), and CD8 T cells (d) separately. CGD: chronic granulomatous disease (n=5), FMF: familial Mediterranean fever (n=7), gout (n=6) FMF patient who also has Behcet disease is indicated in lighter color. Non-parametric Mann-Whitney test was applied for statistical analysis. Supplementary Figure 3 Representative dot plots showing gating strategy for CD38 (a) and CD69 (b) on Vδ2, CD4 and CD8 T cells in freshly isolated PBMCs. Supplementary Figure 4 Representative dot plots showing gating strategy for CD95 (a) and exhaustion markers PD1 (b) and CTLA-4 (c) on Vδ2, CD4 and CD8 T cells in freshly isolated PBMCs. Supplemen [file 10875_2023_1576_MOESM4_ESM.pdf]

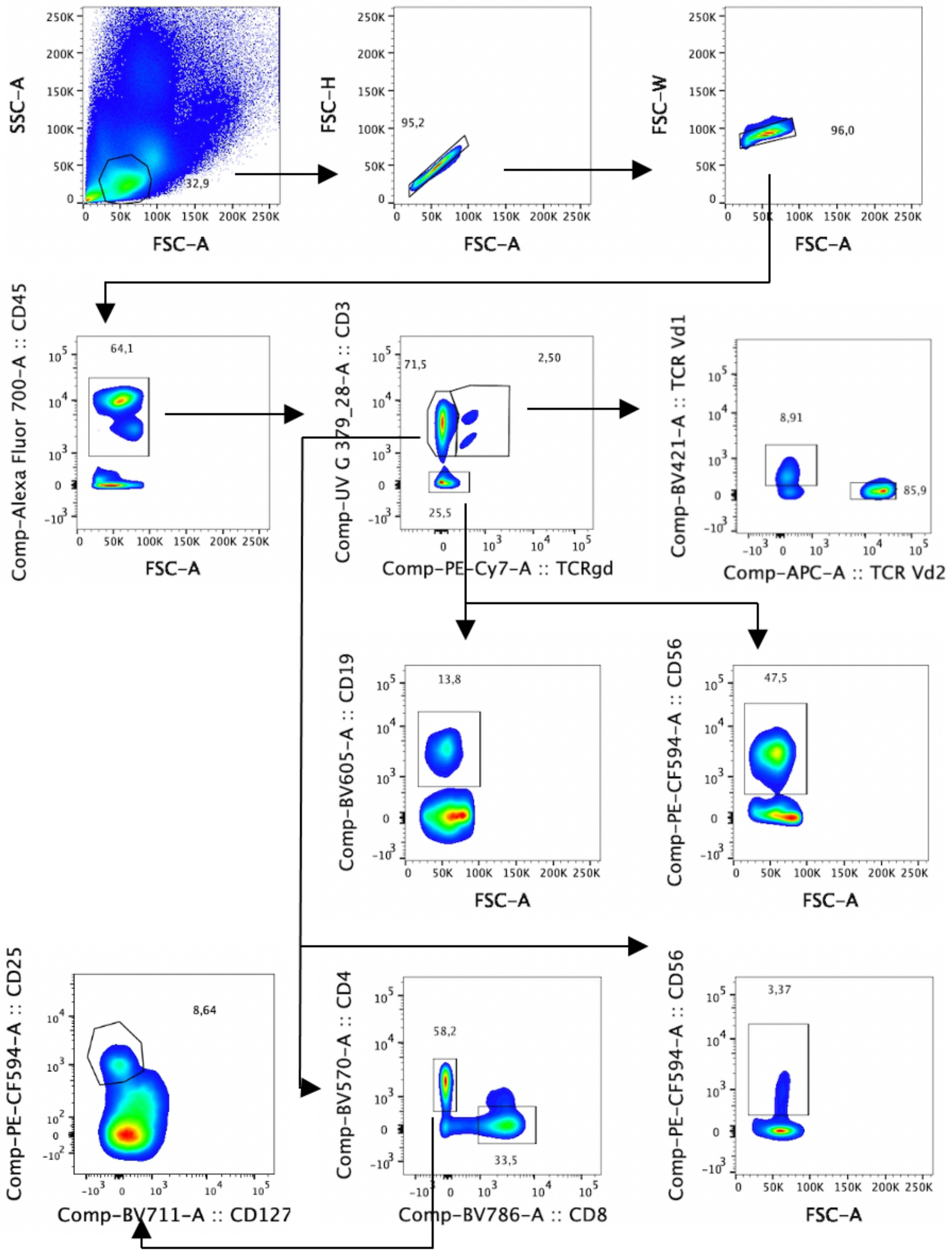

Supplementary Figure 1

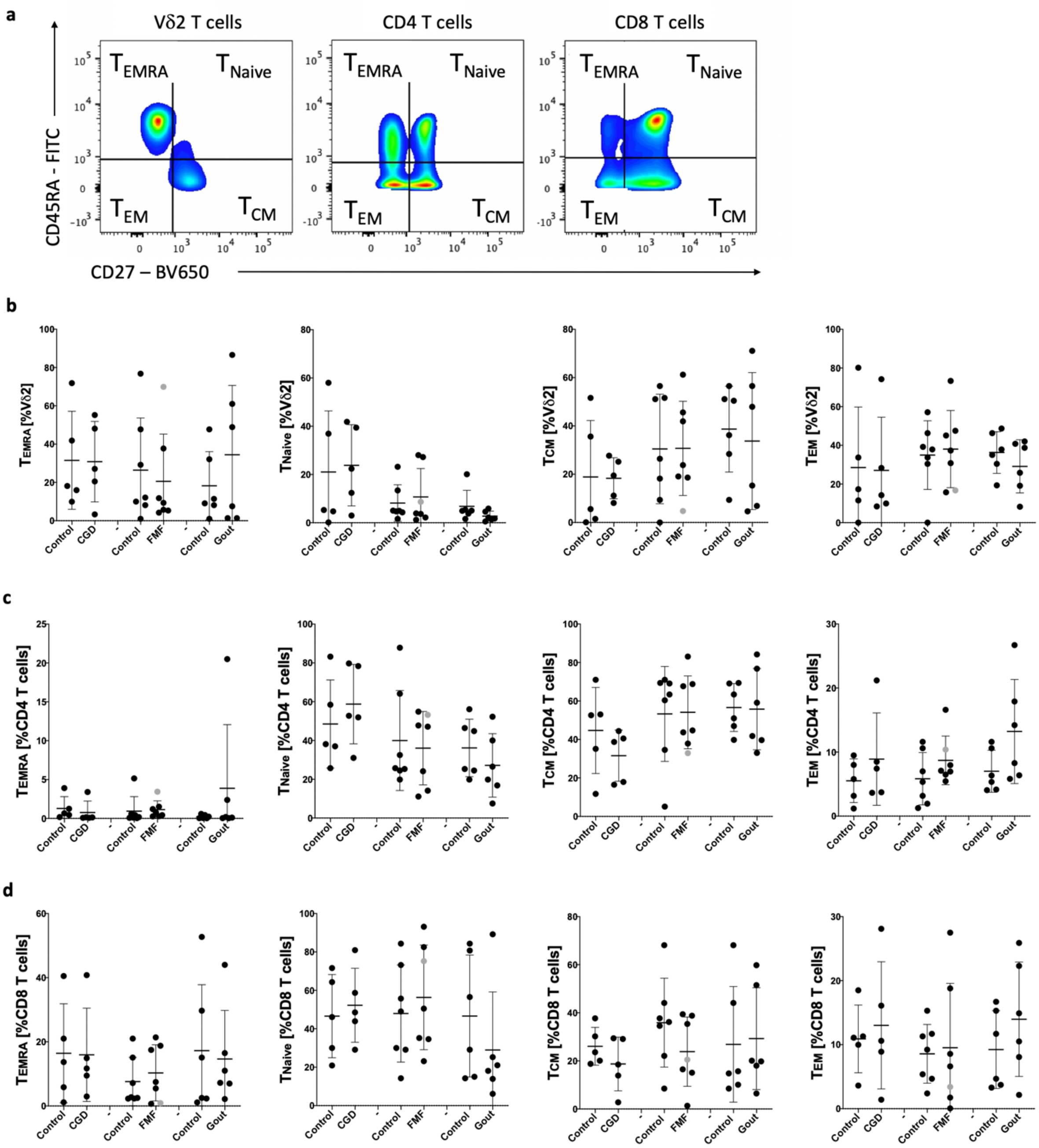

Supplementary Figure 2

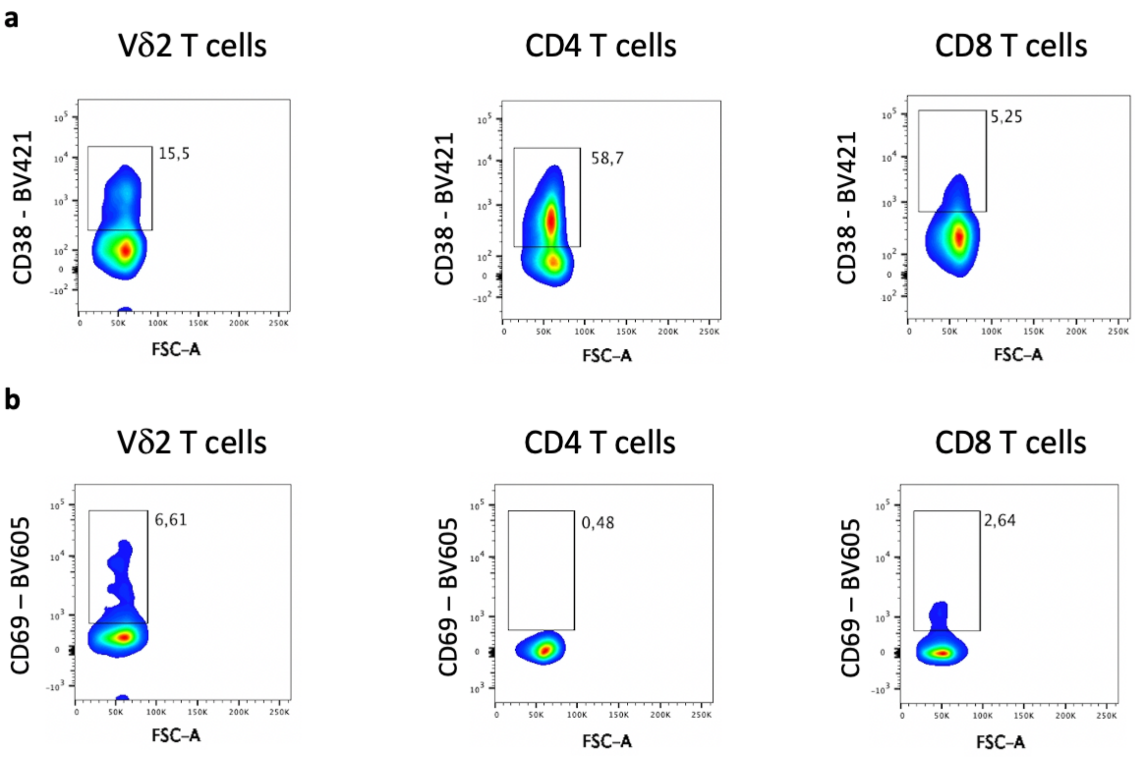

Supplementary Figure 3

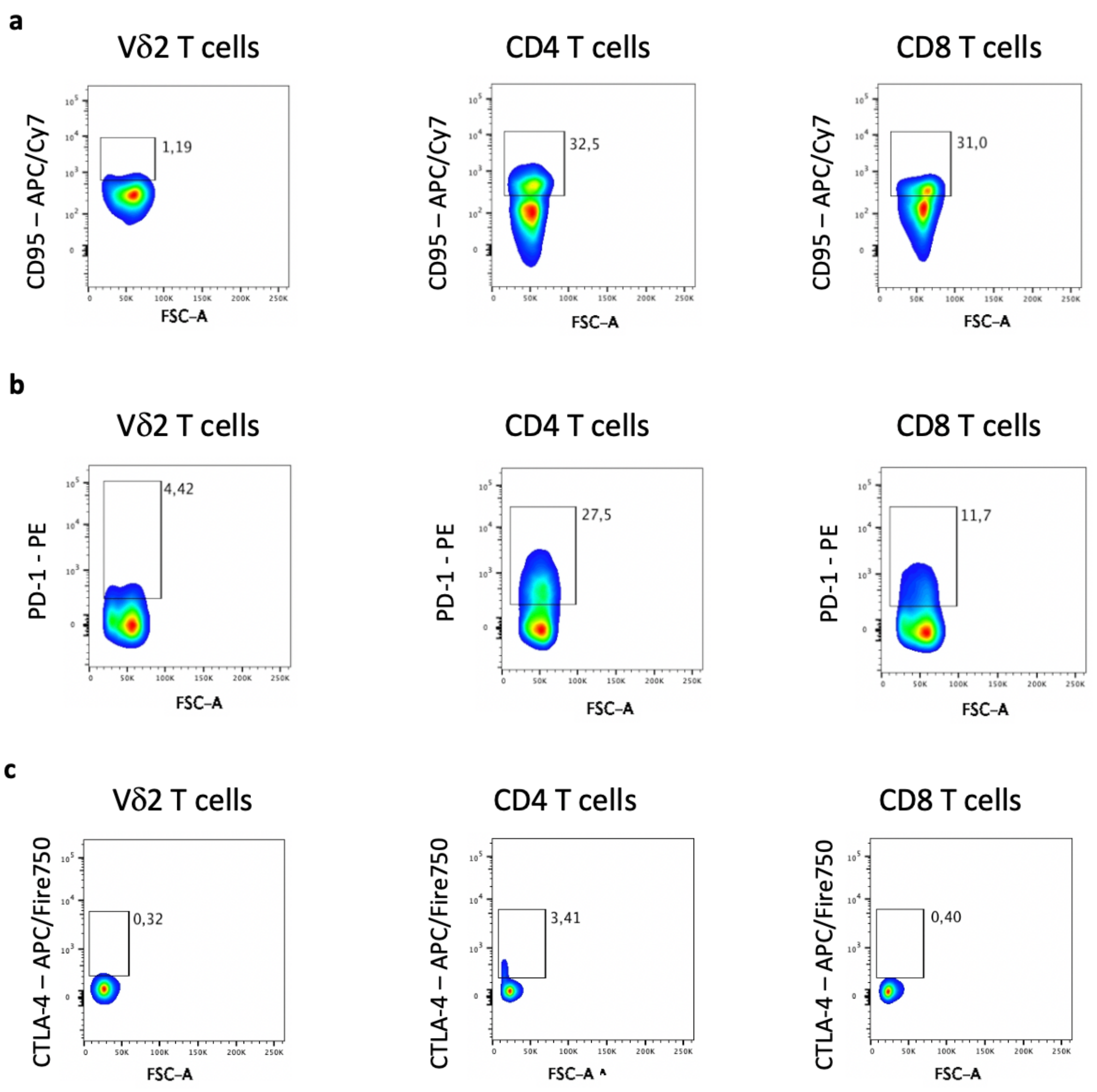

Supplementary Figure 4

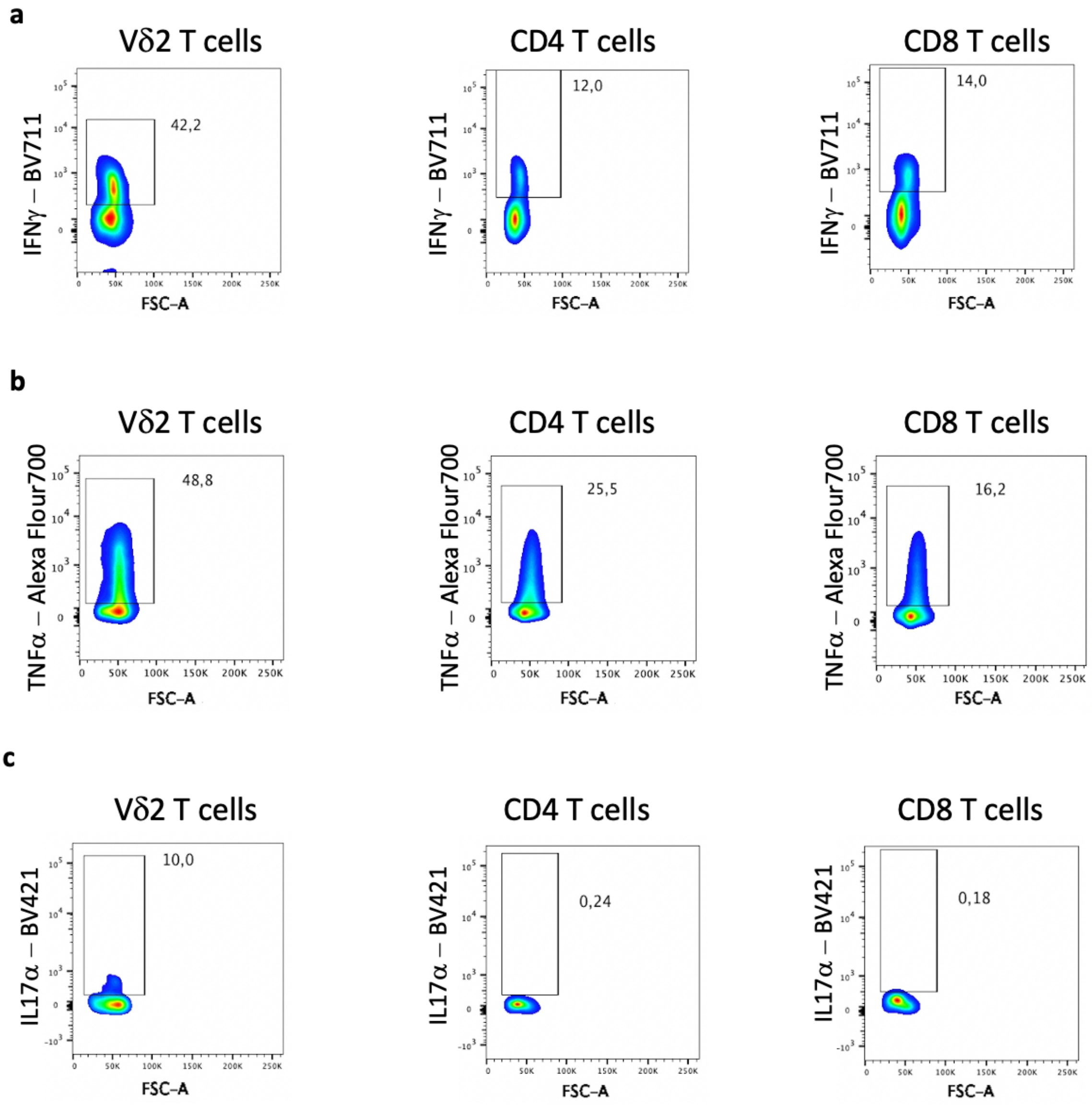

Supplementary Figure 5

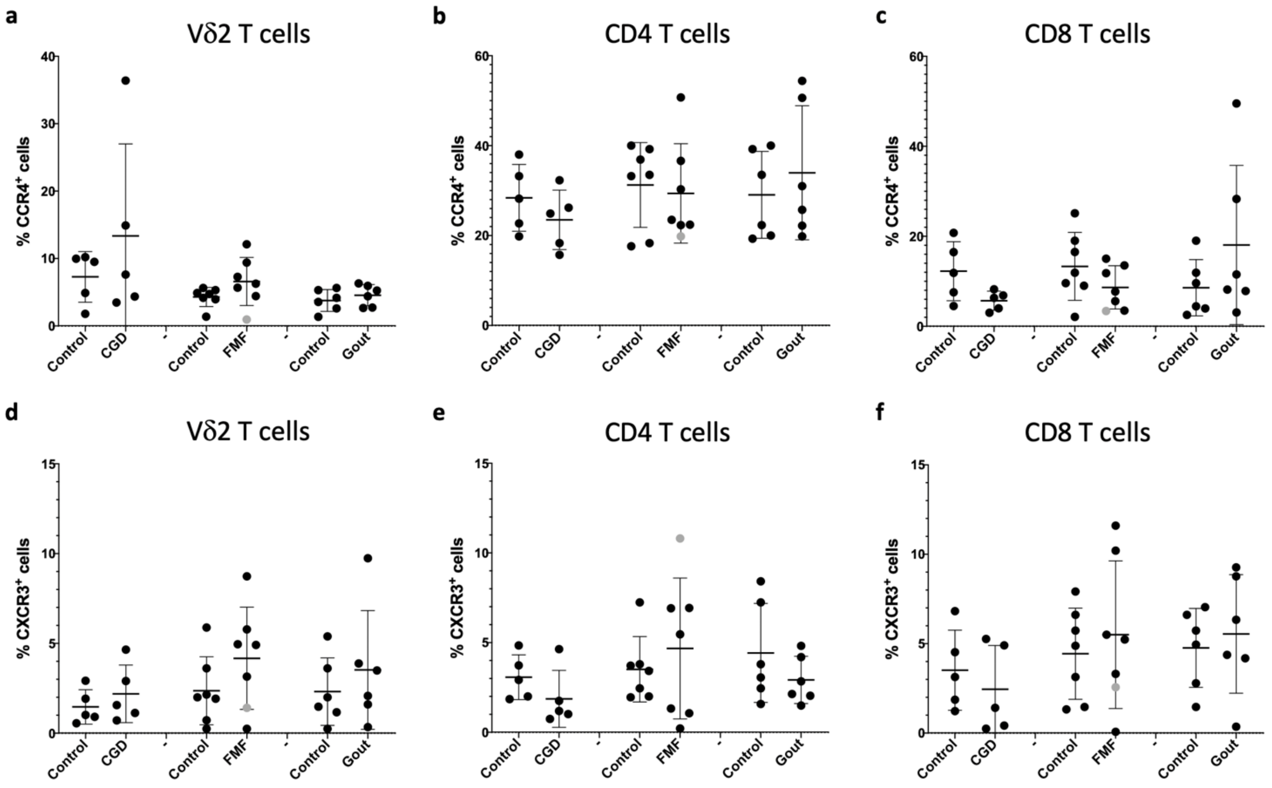

Supplementary Figure 6

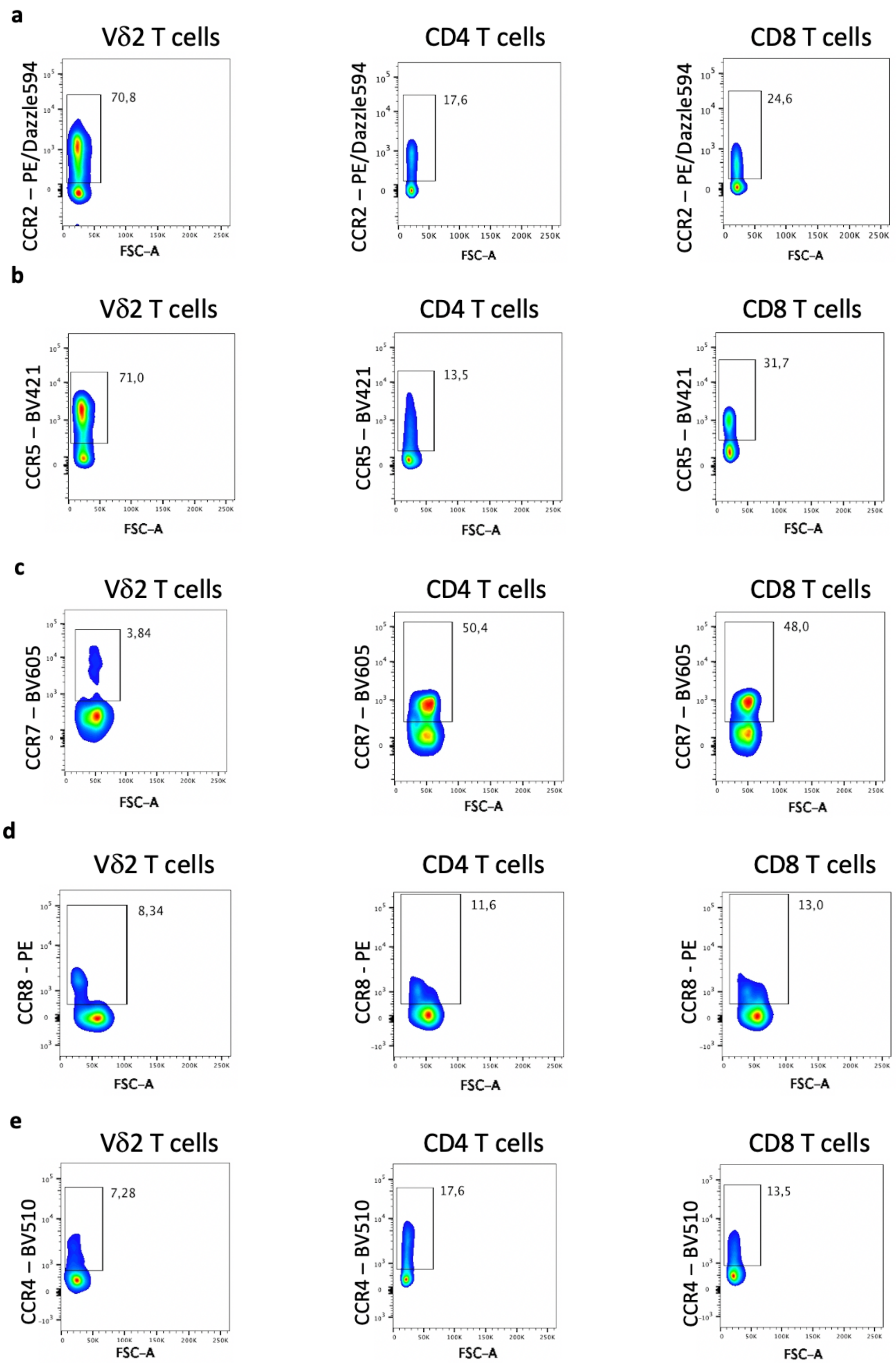

a
Vδ2 T cells
CD4 T cells
CD8 T cells
CCR2 – PE/Dazzle594
FSC-A
70,8
17,6
24,6
b
Vδ2 T cells
CD4 T cells
CD8 T cells
CCR5 – BV421
FSC-A
71,0
13,5
31,7
c
Vδ2 T cells
CD4 T cells
CD8 T cells
CCR7 – BV605
FSC-A
3,84
50,4
48,0
d
Vδ2 T cells
CD4 T cells
CD8 T cells
CCR8 - PE
FSC-A
8,34
11,6
13,0
e
Vδ2 T cells
CD4 T cells
CD8 T cells
CCR4 – BV510
FSC-A
7,28
17,6
13,5

f

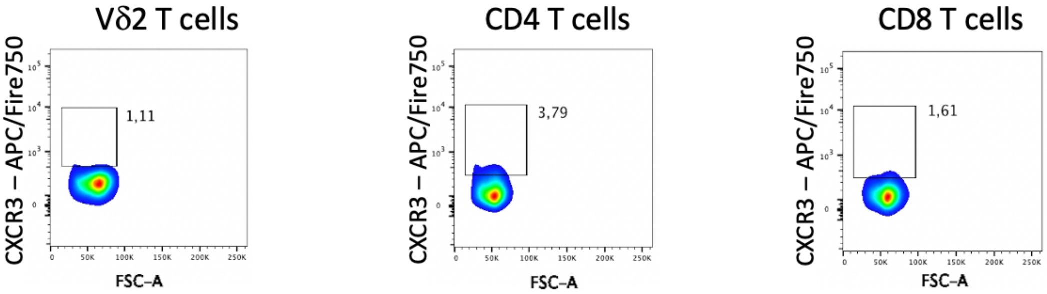

Supplementary Figure 7

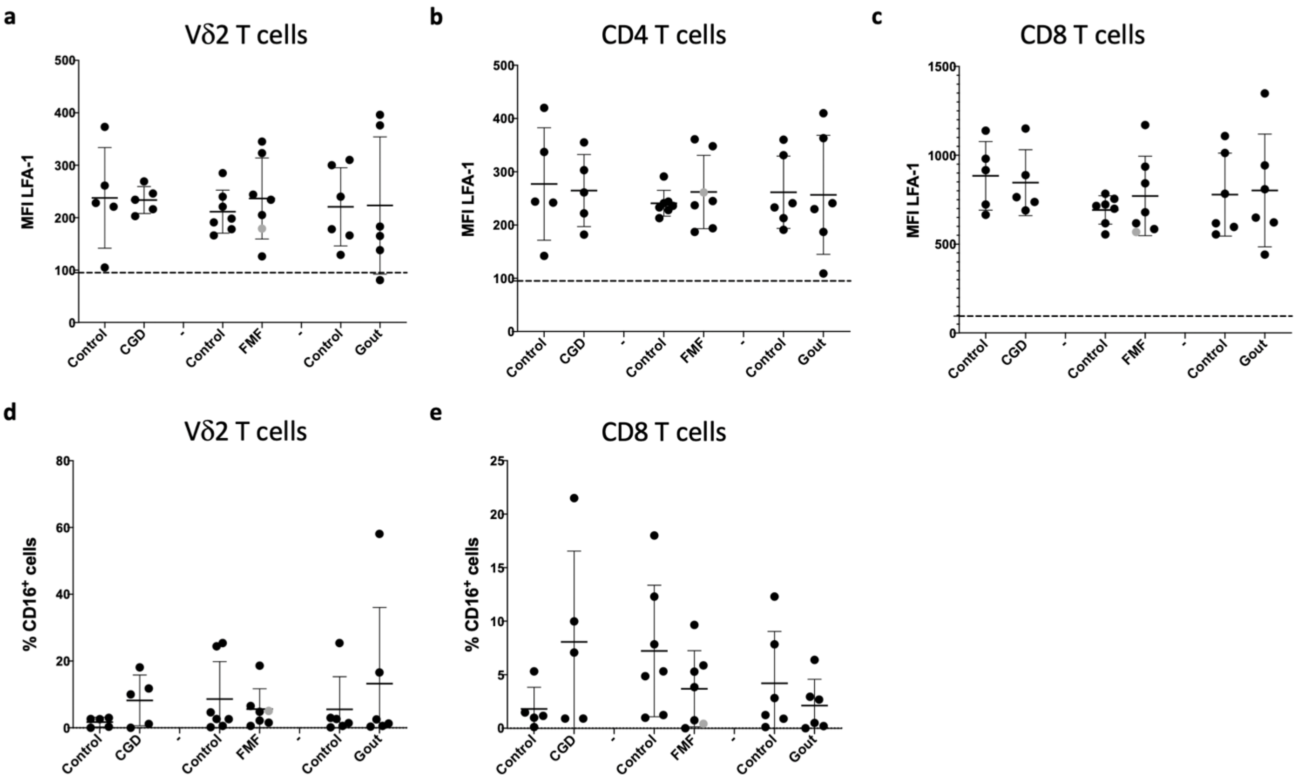

Supplementary Figure 8

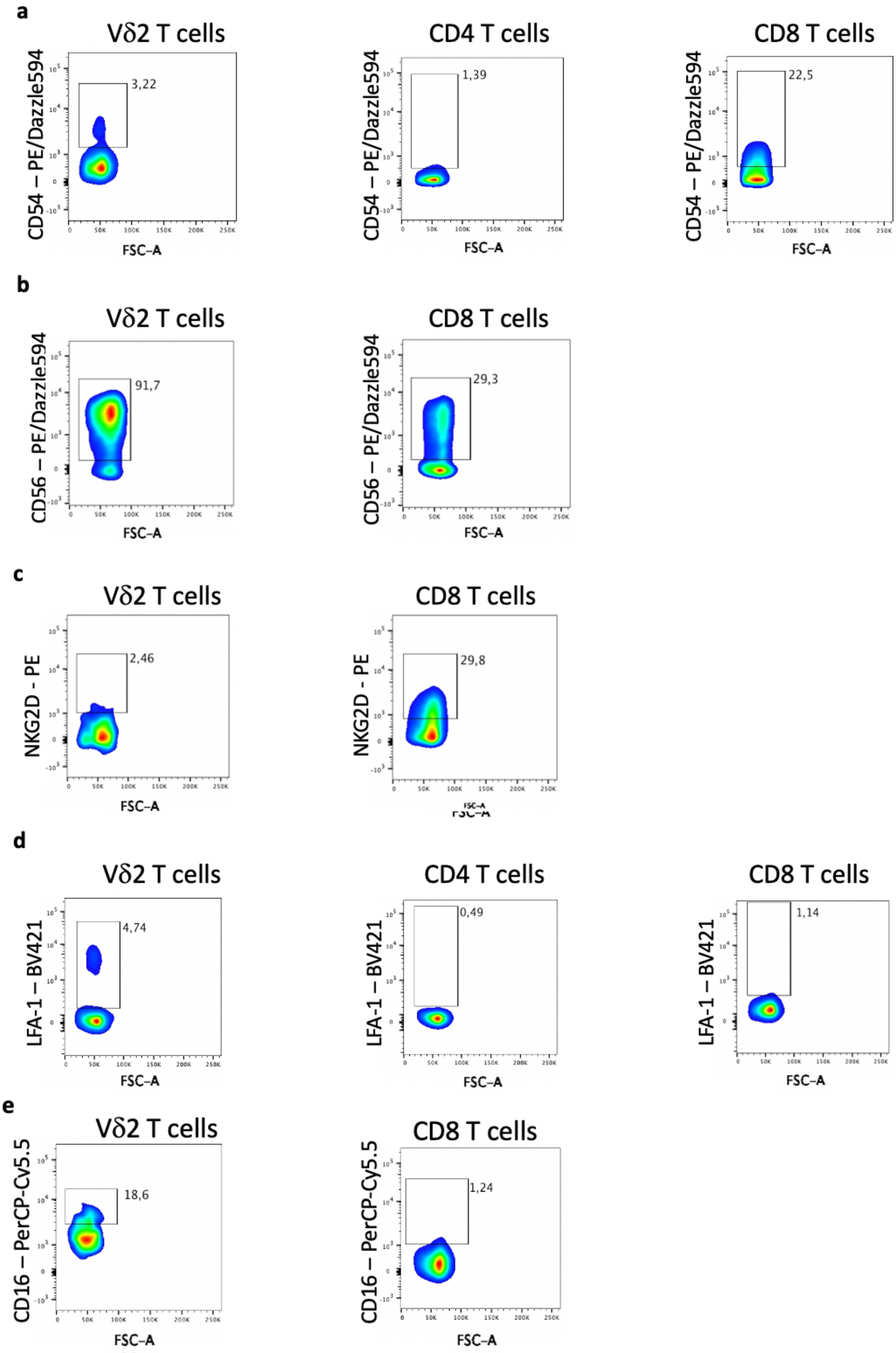

Supplementary Figure 9
